# E2F3 activates NF-κB signaling through TRIM26 mediated TAB1 ubiquitination in pancreatic cancer

**DOI:** 10.7150/ijbs.127710

**Published:** 2026-04-08

**Authors:** Qiyue Zhang, Boping Jing, Dianyun Ren, Yan Sun, Hongkun Cai, Xueyi Liang, Yingke Zhou, Heshui Wu, Feng Guo

**Affiliations:** 1 Department of Pancreatic Surgery, Union Hospital, Tongji Medical College, Huazhong University of Science and Technology, Wuhan 430022, China.; 2 Sino-German Laboratory of Personalized Medicine for Pancreatic Cancer, Union Hospital, Tongji Medical College, Huazhong University of Science and Technology, Wuhan 430022, China.; 3 Department of Nuclear Medicine, Union Hospital, Tongji Medical College, Huazhong University of Science and Technology, Wuhan, 430022, China.; 4 Department of Thoracic Surgery, Union Hospital, Tongji Medical College, Huazhong University of Science and Technology, Wuhan, 430022, China.

**Keywords:** PDAC, E2F3, NF-κB pathway, TRIM26, K11 ubiquitination

## Abstract

Pancreatic ductal adenocarcinoma (PDAC) remains one of the deadliest cancers with limited therapeutic options. Dysregulated transcriptional networks are key drivers of its aggressive biology. Here, by integrating clinical datasets with mechanistic studies, we performed a family wide systematic analysis of E2F transcription factors and identified E2F3 as a key oncogenic driver with prognostic significance comparable to E2F1. Functional studies showed that E2F3 accelerates PDAC proliferation and xenograft growth. Mechanistically, E2F3 transcriptionally activates the E3 ligase TRIM26, which binds TAB1, promotes K11-linked polyubiquitination, and facilitates TAB1-TAK1 complex formation to engage canonical NF-κB signaling. The SPRY and RING domains of TRIM26 mediate TAB1 interaction and ubiquitination, respectively. TRIM26 depletion attenuated E2F3 induced NF-κB activation and tumor growth, whereas its restoration rescued these effects. Clinically, E2F3, TRIM26, and phosphorylated p65 levels were positively correlated in PDAC tissues, and therapeutic delivery of siTRIM26 recapitulated NF-κB inhibition. These findings uncover an unrecognized E2F3-TRIM26-TAB1/TAK1-NF-κB signaling axis that links cell cycle regulation with inflammatory activation in PDAC and nominate TRIM26 as a druggable vulnerability to therapeutically decouple this oncogenic crosstalk.

## Introduction

Pancreatic ductal adenocarcinoma (PDAC) is still one of the deadliest cancers^1^. Current treatments such as FOLFIRINOX^2^ or nab-paclitaxel with gemcitabine^3^, and even immunotherapy^4^, ^5^, have brought only limited survival benefits. These realities underscore the need to define tumor intrinsic drivers that couple proliferative programs to inflammatory signaling, thereby revealing tractable points for intervention. In PDAC, such biology is shaped by deregulated cell cycle control and persistent NF-κB activity^6^, ^7^.

The E2F transcription factor family (E2F1-E2F8) governs the G1/S transition downstream of the RB pathway^8^ and is frequently deregulated in PDAC owing to alterations such as CDKN2A loss^9^. Beyond cell cycle control, E2Fs influence DNA replication^10^ and stress responses^11^, and can interface with inflammatory circuits. However, most investigations have centered on E2F1^12^, ^13^, whereas the roles of other members remain less defined, and at times context dependent^14^, ^15^. Notably, E2F3 is an established oncogenic factor in several cancers^16^-^18^, yet it has not been systematically evaluated in PDAC or linked mechanistically to inflammatory signaling. These considerations highlight two gaps: (i). PDAC lacks a clinically anchored appraisal of the E2F family to determine which member beyond the well studied E2F1 carry the greatest adverse prognostic weight; (ii). whether a specific E2F engages NF-κB through a defined upstream module is unknown.

To address these gaps, we began with a family wide analysis across multiple PDAC cohorts and applied multivariable survival modeling to prioritize candidates by prognostic impact. This approach highlighted E2F3, whose association with poor outcome is comparable to E2F1 yet whose function in PDAC is underexplored. Guided by this clinical prioritization, we demonstrate that E2F3 is consistently upregulated in PDAC and functions as a potent driver of tumor growth. Mechanistically, our analyses identify TRIM26 as a direct transcriptional target of E2F3, establishing a link between cell cycle control and ubiquitin signaling. Through regulation of TAB1 ubiquitination and stabilization of the TAB1-TAK1 complex, TRIM26 amplifies canonical NF-κB activation, thereby reinforcing inflammatory survival programs. Functional rescue experiments in cell based and animal models further validate the dependence of PDAC growth on this E2F3-TRIM26-NF-κB axis. Moreover, proof of concept inhibition of TRIM26 using siRNA suppressed PDAC progression, highlighting TRIM26 as a therapeutically accessible node.

Together, these findings uncover a previously unrecognized connection between a cell cycle transcription factor and ubiquitin mediated inflammatory signaling, and provide a mechanistic and clinically informed rationale for targeting the E2F3-TRIM26 pathway to blunt NF-κB-driven tumor progression.

## Materials and Methods

### Cell lines and cell culture

PANC-1, CAPAN-1, and CFPAC-1 cells were cultured in high glucose DMEM (BasalMedia, Cat# L110KJ) supplemented with 10% Certified Premium FBS (Vivacell, Cat# C04001) and 1% antibiotic antimycotic (BasalMedia, Cat# S120JV). ASPC-1 and BXPC-3 cells were maintained in RPMI-1640 (BasalMedia, Cat# L210KJ) with 10% FBS and 1% antibiotic antimycotic. SW1990 cells were cultured in Leibovitz's L-15 medium (BasalMedia, Cat# L620KJ) with 10% FBS and 1% antibiotic antimycotic and incubated at 37 °C in ambient air without CO₂. All cell lines were maintained at 37 °C.

### Western blot analysis

Cells were lysed on ice for 30 min in RIPA buffer containing protease and phosphatase inhibitors. Clarified lysates (12,000 rpm, 15 min, 4 °C) were quantified (BCA), resolved by SDS-PAGE, and transferred to PVDF membranes. Membranes were blocked in 5% non fat milk for 1 h at room temperature, incubated with primary antibodies overnight at 4 °C, washed with TBST, and probed with HRP conjugated secondary antibodies for 1 h at room temperature. Signals were developed using ECL and captured on digital imager. Primary antibodies used in this study are summarized below and detailed in Supplementary Table S1. Housekeeping controls included GAPDH.

### Immunohistochemistry (IHC)

Commercial PDAC tissue microarrays (Avilabio, Shaanxi, China; D049Pa01; 49 cases, including 45 tumor tissues and 4 normal pancreatic tissues) were processed using standard IHC protocols. Primary antibodies included E2F3, TRIM26, and phosphorylated p65. Slides were independently scored by two board certified pathologists blinded to clinical data. Staining intensity was graded 0-3 (0, negative; 1, light yellow; 2, dark yellow; 3, brown). The percentage of positive cells was scored 1-4 (1, 1-25%; 2, 26-50%; 3, 51-75%; 4, 76-100%). The final IHC score equaled the sum of the two components.

### Quantitative RT-qPCR

Total RNA was extracted with TRIzol (Invitrogen), reverse transcribed using PrimeScript (Takara), and quantified by SYBR based qPCR (TB Green, Takara) according to the manufacturers' instructions. GAPDH served as the internal reference. Relative expression was calculated by the 2^-ΔCt method. Primer sequences are provided in Supplementary Table S2.

### RNA interference

Predesigned TRIM26 shRNA target sequences were obtained from MilliporeSigma (TRC collection). Oligonucleotides were cloned into pLKO.1-puro under the U6 promoter using AgeI/EcoRI sites according to the TRC protocol. Lentiviral particles were produced in HEK-293T cells with pLKO.1-shRNA. Full shRNA sequences are listed in Supplementary Table S3. For transient silencing, synthetic siRNAs targeting STYX (5′-GAGGCCCAGAGTGTGTATACC-3′) was transfected using Lipofectamine RNAiMAX (Thermo Fisher) according to the manufacturer's protocol.

### Plasmids and transient transfection

The plasmids E2F3 and HA-ubiquitin (including wild type and linkage restricted variants) were obtained from GENECHEM. Human TRIM26 with a C-terminal Myc tag, along with the deletion mutants ΔRING (aa 16-57) and ΔSPRY (aa295-539), were cloned into pcDNA™3.1/myc (Invitrogen, V800-20) using the XhoI and EcoRI sites. TAB1 with a C-terminal Flag tag was constructed in pcDNA3.1-Flag (Addgene 208051) via NheI and ApaI restriction sites. Plasmids were transfected into cells using Lipofectamine 3000 (Thermo Fisher) according to the manufacturer's instructions, and cells were harvested 48 h post transfection.

### Functional assays

Clonogenic survival was assessed by plating 1,500 cells per well in 6 well plates and culturing for 14 days, followed by fixation with 4% paraformaldehyde and staining with 0.1% crystal violet. Cell migration was evaluated by wound healing assays, in which confluent monolayers were scratched with a sterile pipette tip, cultured in serum free medium, and imaged at indicated time points. Transwell migration assays were performed using 10,000 cells seeded in serum free medium into the upper chamber, with medium containing 30% FBS in the lower chamber; migrated cells were fixed, stained with crystal violet, and counted microscopically. For CCK-8 assays, 5,000 cells per well were seeded in 96 well plates and cultured for up to 5 days; cell viability was measured daily using the Cell Counting Kit-8 (Beyotime, Cat# C0037) according to the manufacturer's instructions.

### Chromatin immunoprecipitation (ChIP) and ChIP-qPCR

ChIP was performed with the Abcam Chromatin Extraction kit (ab117152) and One Step Magnetic ChIP kit (ab156907) according to the instructions, using antibodies against E2F3 (with normal IgG as control). Purified ChIP DNA and 1% input were quantified by real time PCR with TB Green. Enrichment was calculated relative to input and IgG. Primer sequences are provided in Supplementary Table S4.

### CUT&Tag (Cleavage Under Targets and Tagmentation)

CUT&Tag was performed using the Hyperactive Universal CUT&Tag Assay Kit for Illumina (Vazyme, Cat# TD903) according to the manufacturer's protocol. Briefly, 5 × 10⁴ cells were immobilized on ConA coated beads, incubated with primary antibodies overnight at 4 °C, followed by secondary antibody and pA/G-Tn5 transposome binding. Tagmentation was carried out at 37 °C, and DNA was purified using magnetic beads. Libraries were amplified and sequenced on an Illumina platform.

### RNA-seq

Total RNA was isolated with TRIzol (Invitrogen, #15596026). Library construction and sequencing were performed by Novogene (Beijing, China). Indexed libraries were prepared according to the vendor's protocol and sequenced on an Illumina NovaSeq platform to generate 150 bp paired end reads.

### Co-immunoprecipitation (Co-IP) and Ubiquitination assay

Cells were lysed in Beyotime IP buffers (P0013) supplemented with protease/phosphatase inhibitors. Clarified lysates were subjected to immunoprecipitation with anti-Myc (TRIM26) or anti-Flag (TAB1), with normal IgG as control. Input fractions were retained. Immunoprecipitates and inputs were analyzed by Western blotting to detect TAB1, TAK1, TRAF6, TRIM26, and epitope tags as indicated.

For TAB1 polyubiquitination, cells were co-transfected with Flag-TAB1 and HA-ubiquitin (WT or linkage mutants). six hours before harvest, cells were treated with 10 μM MG132 (Beyotime Y210207). Cells were lysed and boiled (10 min), and diluted 10× with NP-40 buffer. TAB1 was immunoprecipitated with anti Flag, washed, and subjected to immunoblotting with anti-HA.

### Luciferase reporter assay and site-directed mutagenesis

TRIM26 promoter fragment (-400 to +100 bp relative to the TSS) was PCR amplified from human genomic DNA and cloned into pGL3-Basic (Addgene #212936) using KpnI/HindIII, generating pGL3-TRIM26-WT. The putative E2F3 motif was disrupted by site-directed mutagenesis (Vazyme, Mut Express II Fast) converting bases 353-357 (GGCGC → AACAC) to yield pGL3-TRIM26-Mut. Primer sequences used for promoter cloning and mutagenesis are provided in Supplementary Table S5.

For reporter assays, PANC-1 cells in 24-well plates were co-transfected with 400 ng of pGL3-TRIM26-WT or pGL3-TRIM26-Mut and 200 ng of pCMV-E2F3 using Lipofectamine 3000 (Thermo Fisher). pRL-TK (40 ng/well; Addgene #27163) served as the Renilla control. At 36 h, cells were lysed and Firefly/Renilla activities were measured with the Dual-Luciferase Reporter Assay (Beyotime, RG027). Relative promoter activity was calculated as the Firefly/Renilla ratio.

### Animal experiments

Female BALB/c nude mice (4-6 weeks old) were purchased from Vitalriver (Beijing, China) and maintained under SPF (specific pathogen-free) conditions with free access to food and water. For xenograft studies, PANC-1 cells (1 × 10⁷) were resuspended in 50 μL PBS and mixed with an equal volume of growth factor reduced Matrigel (Corning, USA) on ice. A total of 100 μL of the cell suspension was subcutaneously injected into the flank region of each mouse. Tumor size was measured every two days with digital calipers, and volume was calculated as (L × W^2)/2. For therapeutic intervention studies, mice bearing established xenografts were randomly assigned to three groups. Chemically stabilized siTRIM26 (5′-cholesterol conjugated, fully 2′-O-methyl modified; RiboBio, Guangzhou, China) was administered at 1 mg/kg by intravenous injection twice weekly, starting on day 10 after implantation and continuing for two weeks. NF-κB pathway inhibitor BAY 11-7082 (15 mg/kg) was delivered intraperitoneally every other day (QOD). Animals were sacrificed on day 25 or when tumors reached the humane endpoint. All animal experimental procedures were performed according to guidelines set forth by the Chinese National Institutes of Health and approved by the Ethical Committee on Animal Experiments of the Huazhong University of Science and Technology in Wuhan, China (IACUC approval No. [2023]4405).

### Bioinformatics and statistical analyses

Public datasets and preprocessing Transcriptomic and clinical data for pancreatic adenocarcinoma (PAAD) were downloaded from TCGA; normal pancreatic expression profiles were obtained from GTEx. Gene counts/FPKM were converted to TPM and log2 transformed for downstream analyses; samples without clinical or survival data were excluded. Independent validation cohorts were retrieved from GEO (GSE15471, GSE16515, GSE28735, GSE71729, GSE62452).

In house RNA-seq processing: Raw reads were trimmed and aligned to GRCh38 using STAR. Gene level counts were summarized with featureCounts. Differential expression was performed with DESeq2; significance criteria were FDR < 0.05 and |log2FC| > 1. Heatmaps and volcano plots were generated with pheatmap and ggplot2.

Pathway enrichment and GSEA: Functional annotation of DEGs (Differentially Expressed Genes) used clusterProfiler for GO (Gene Ontology) and KEGG (Kyoto Encyclopedia of Genes and Genomes). Gene set enrichment analysis (GSEA) was performed on log2FC ranked lists (functions gseGO, gseKEGG) with gene sets from org.Hs.eg.db and KEGG; multiple testing correction used BH FDR. To generate the family-level enrichment overview, the eight E2F family members were used as the input gene set for KEGG and Gene Ontology enrichment analyses to summarize the major functional and pathway associations of the E2F family.

E2F3 target nomination and promoter analysis: Putative E2F3 targets were retrieved from hTFtarget. The TRIM26 promoter (-2000 to +100 bp relative to TSS) was extracted from Ensembl BioMart/UCSC (hg38). JASPAR2022 motifs (E2F3 PWM, MA0472.3) were scanned with motifmatchr using a relative profile score ≥ 0.70 and complementary p value threshold p < 1×10⁻⁴. Predicted TFBS were annotated with TFBSTools.

ChIP external data: Public E2F3 ChIP-seq (GSM1239452) was downloaded and visualized with IGV to corroborate binding at the TRIM26 promoter and other loci.

Correlation and co-expression: Pearson correlations were computed on log2(TPM+1) values in TCGA-PAAD to assess associations between E2F3, TRIM26, NF-κB pathway genes, and cytokines. Scatter plots and correlation matrices were produced with ggstatsplot.

Survival and prognostic modeling: Overall survival, disease specific survival, and progression-free interval were analyzed using Cox proportional hazards models (univariate and multivariable) with age, sex, T stage, and histologic grade as covariates. Kaplan-Meier curves were drawn with survminer. A nomogram based on the multivariable Cox model was built with rms; performance was assessed by Harrell's C index. Stratified Cox models were visualized as forest plots with ggplot2 (R 4.2.1).

## Results

### E2F3 is one of the top prognostic candidates among E2F family members in PDAC

**Figure 1:**
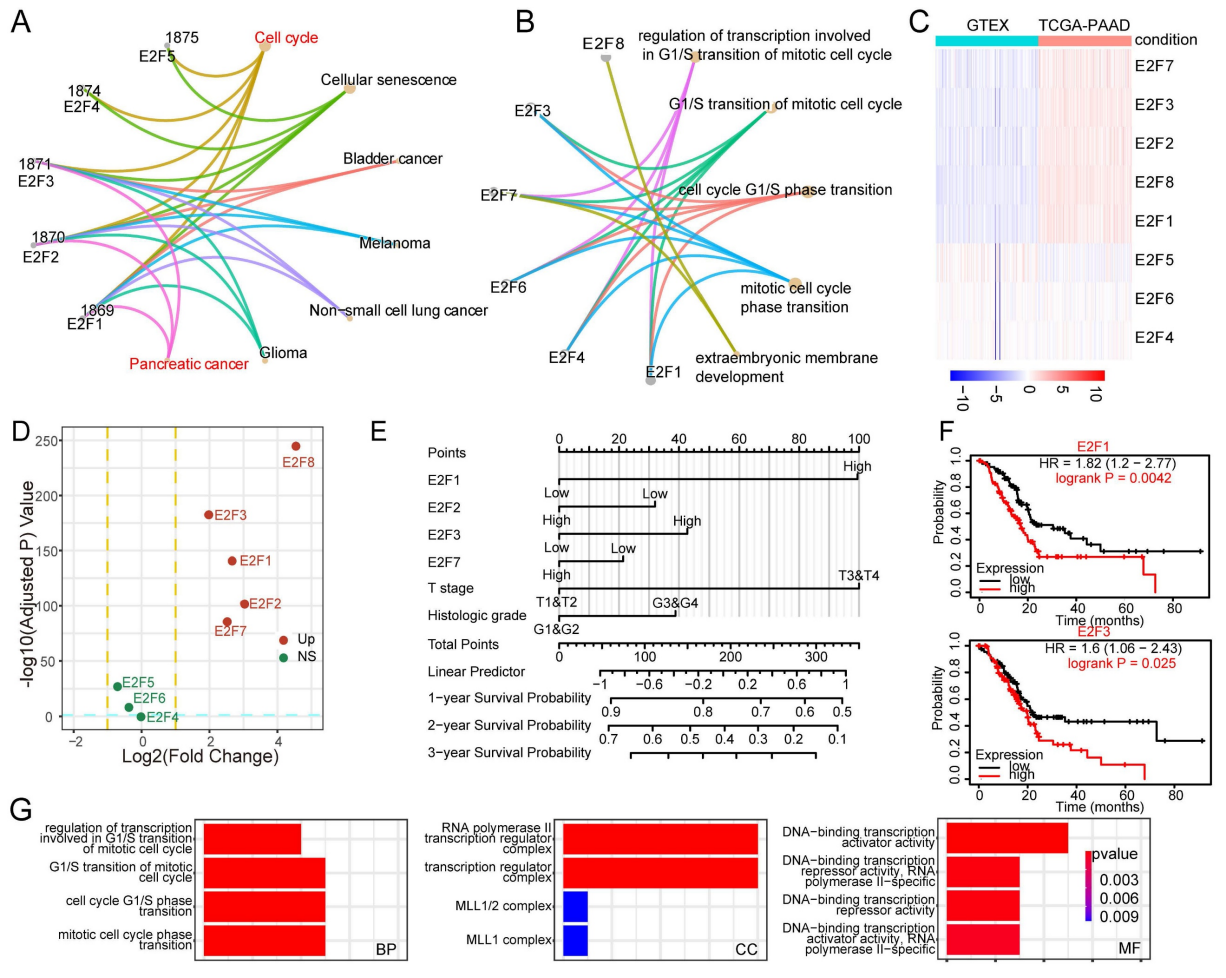
Identification of E2F3 as a prognostic driver within the E2F family in pancreatic cancer. (A) KEGG (Kyoto Encyclopedia of Genes and Genomes) chord diagram mapping E2F family members to enriched pathways and cancer types; E2F1-3 are jointly enriched in cell cycle processes and pancreatic cancer. (B) GO (Gene Ontology) biological process enrichment of E2Fs highlighting mitotic cell cycle, G1/S transition, and transcriptional regulation. (C) Heatmap of E2F family expression in TCGA-PAAD (tumor vs. normal), showing upregulation of E2F1, E2F2, E2F3, E2F7, and E2F8. (D) Volcano plot of TCGA-PAAD comparing tumor vs. normal samples; significantly dysregulated E2F genes are labeled. (E) Cox based prognostic nomogram integrating E2F1/2/3/7/8 with clinicopathologic variables to estimate survival risk. (F) Kaplan-Meier curves for overall survival stratified by median expression showing worse outcomes for E2F1 high and E2F3 high patient. (G) GO enrichment of differentially expressed genes between top vs. bottom E2F3 expression quartiles in PDAC, revealing programs in cell cycle progression, DNA replication, and chromatin organization.

To delineate the biological significance of the E2F transcription factor family in PDAC, we performed pathway enrichment analysis using KEGG and GO gene sets. The KEGG chord diagram revealed that several E2F members, particularly E2F1, E2F2, and E2F3, were simultaneously enriched in cell cycle regulation and pancreatic cancer, underscoring their joint contribution to tumor initiation (Figure 1A). GO analysis further indicated that E2F factors are mainly involved in mitotic cell cycle regulation and G1/S transition, consistent with their roles as core cell cycle regulators (Figure 1B). These findings suggest of E2F1, E2F2 and E2F3 in driving pancreatic tumorigenesis. To determine which E2F members may be functionally relevant in PAAD, we analyzed gene expression patterns from TCGA. Heatmap and volcano plot analyses revealed that E2F1, E2F2, E2F3, E2F7, and E2F8 were significantly upregulated in pancreatic tumors compared with normal tissues (Figure 1C-D).

We next assessed the prognostic relevance of E2F family members in PDAC using Cox regression analysis. Gene expression values were dichotomized into high and low groups based on median expression. In univariate analysis, high expression of E2F1, E2F2, E2F3, E2F7, and E2F8, as well as advanced T stage, were associated with worse overall survival (p < 0.1; Table 1). These variables were subsequently included in multivariate analysis. The results confirmed that E2F1 (HR (hazard ratio) = 2.002, 95% CI: 1.082-3.703, p = 0.027) and T stage (HR = 2.009, 95% CI: 1.022-3.950, p = 0.043) served as independent prognostic factors. While E2F3 did not reach statistical significance (HR = 1.347, p = 0.185), its elevated hazard ratio suggests a potential role in disease progression, potentially masked by overlapping effects with other clinical variables.

To further assess the prognostic relevance of the E2F family, we constructed a Cox regression-based nomogram that integrated gene expression profiles with clinical variables. Among the four candidate genes, E2F1 emerged as the strongest predictor of poor survival, with E2F3 ranking second, suggesting that E2F3 may play an important role in PDAC progression (Figure 1E). In contrast, E2F2 showed a modest inverse association with mortality risk, consistent with previous reports describing its protective role in maintaining genomic stability^19^, ^20^. Survival analysis using Kaplan-Meier curves further validated that high expression of E2F1 and E2F3 was significantly associated with poor overall survival in PDAC patients (Figure 1F, Figure S1A-B). To gain mechanistic insight, we stratified PDAC tumors by E2F3 expression and compared global transcriptomic profiles. GO enrichment revealed that E2F3 high tumors were characterized by enhanced cell cycle activity, DNA replication, and chromatin organization (Figure 1G). These proliferative programs highlight E2F3 as a potential oncogenic driver in PDAC.

Collectively, these results position E2F3 as the second most impactful E2F member after E2F1, suggesting it may exert a critical role in promoting pancreatic cancer progression. Given the extensive literature on E2F1, we focused subsequent investigations on defining the functional and mechanistic contributions of E2F3.

### E2F3 upregulation marks aggressive disease and predicts poor prognosis in PDAC

**Figure 2:**
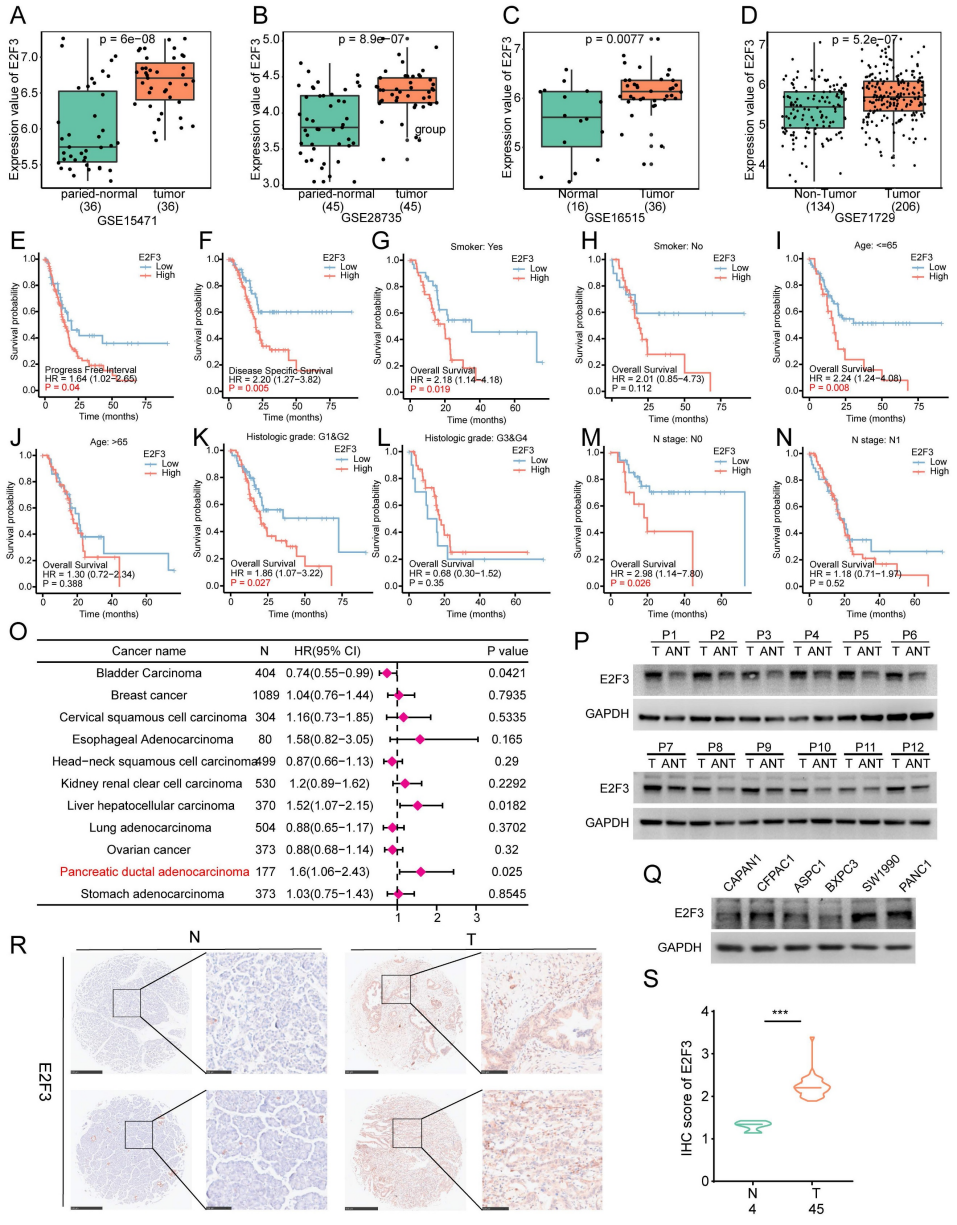
E2F3 is consistently upregulated in PDAC and predicts poor prognosis, especially in early-stage disease. (A-D) E2F3 mRNA is significantly higher in tumor versus matched adjacent tissue across four independent cohorts (GSE15471, GSE28735, GSE16515, GSE71729). (E-N) Stratified Kaplan Meier analyses show worse DSS (disease-specific survival) and PFI (progression free interval) for E2F3 high cases, with the effect retained in smokers, low histologic grade (G1& G2), and early N stage. No significant impact is observed in patients >65 years, high grade, or advanced nodal disease. (O) Pan-cancer Cox forest plot indicating that elevated E2F3 associates with poor prognosis predominantly in hepatocellular carcinoma and PDAC, with the strongest effect in PDAC. (P) Western blot analysis of paired human PDAC specimens confirms higher E2F3 protein expression in tumor (T) relative to adjacent non-tumor (ANT) pancreas. (Q) Baseline E2F3 protein levels across PDAC cell lines. (R) Representative IHC (immunohistochemistry) images of E2F3 in PDAC tumor (T) and normal pancreas (N) tissues from tissue microarrays, scale bars, 500 µm (low magnification) and 100 µm (high magnification). (S) Quantification of E2F3 IHC scores. Statistical analyses were performed using Student's t-test for two-group comparisons, log-rank test for Kaplan-Meier survival analyses, and Cox proportional hazards regression for forest plot analyses. Data are presented as mean ± SD.

To further validate the oncogenic relevance of E2F3 beyond TCGA, we analyzed its expression in four independent pancreatic cancer cohorts (GSE15471, GSE28735, GSE16515, and GSE71729). Consistent results across all datasets confirmed elevated E2F3 expression in PDAC tissues compared to adjacent non tumor tissues (Figure 2A-D).

To examine whether the prognostic effect of E2F3 was consistent across clinical subgroups or influenced by disease context, we performed stratified survival analyses. High E2F3 expression was associated with worse Disease Specific Survival (DSS) and Progression Free interval (PFI), supporting its association with adverse prognosis. Furthermore, in stratified analyses, the deleterious impact of high E2F3 remained evident in subgroups such as smokers, patients with early histologic grade (G1&G2), and those with early N stage, indicating that E2F3 exerts a stronger prognostic effect in earlier stage disease (Figure 2E-N). In contrast, in patients aged > 65, or those with high grade or advanced nodal involvement, E2F3 expression showed no significant effect on survival, possibly due to overriding effects of advanced disease burden. These findings suggest that E2F3 may play a more prominent role in early pancreatic cancer progression.

Forest plot of pan-cancer survival analysis revealed that E2F3 was only associated with poor prognosis in hepatocellular carcinoma and pancreatic adenocarcinoma, with the strongest effect observed in PDAC (Figure 2O). This cancer type specificity may be attributed to KRAS driven oncogenic dependency in pancreatic cancer, where aberrant E2F activation may serve as a key downstream effector^21^, ^22^. Because E2F1 has already been the focus of numerous mechanistic studies in PDAC and other tumors, whereas E2F3 is much less explored despite showing strong and PDAC enriched prognostic power, we considered E2F3 a rational and novel entry point to uncover previously unappreciated signaling circuitry.

To confirm E2F3 expression in clinical samples, we analyzed paired tumor and adjacent tissues from pancreatic cancer patients. WB confirmed elevated E2F3 expression in tumor tissues (Figure 2P). Among several commonly used PDAC cell lines, PANC-1 exhibited the highest E2F3 expression, whereas BXPC-3 showed relatively low levels (Figure 2Q). Immunohistochemistry staining of PDAC microarray further validated strong E2F3 expression in tumor tissues compared to normal pancreas tissues (Figure 2R-S). Collectively, these findings support a consistent role of E2F3 in promoting pancreatic cancer progression.

### E2F3 acts as a key driver of pancreatic cancer cell proliferation and tumor growth

**Figure 3:**
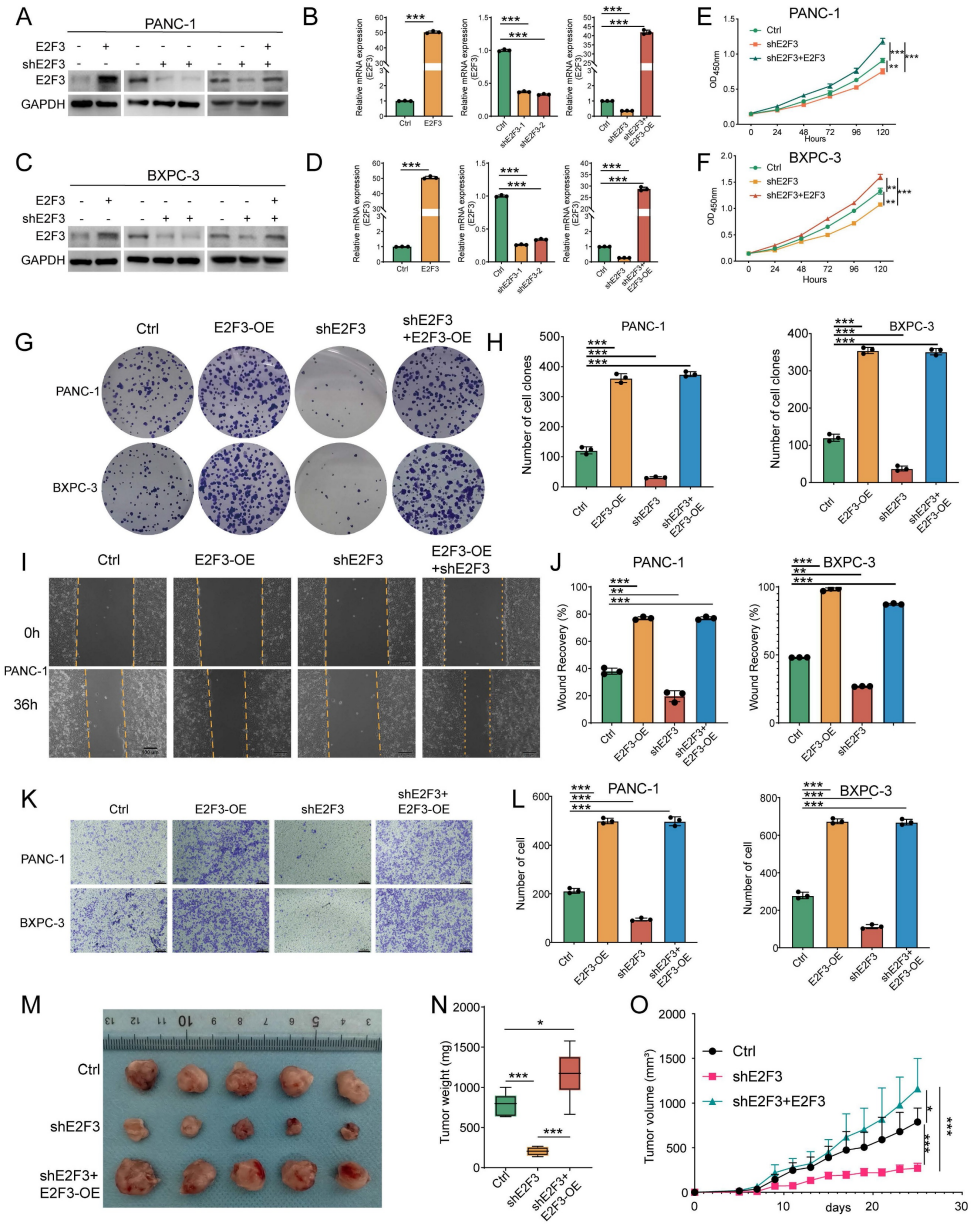
E2F3 promotes PDAC cell proliferation, migration, and tumor growth. (A-B) Immunoblots and qRT-PCR showing efficient modulation of E2F3 expression in PANC-1 cells under four conditions: Ctrl, E2F3-OE (overexpression), shE2F3, and Rescue (shE2F3 + E2F3-OE). GAPDH served as a loading control. (C-D) Immunoblots and qRT-PCR confirming corresponding changes in BXPC-3 cells. (E-F) CCK-8 growth curves showing that E2F3 overexpression accelerates proliferation, knockdown suppresses growth, and rescue restores growth. (G-H) Representative colony formation plates and quantification of single cell colonies for the four groups. (I-J) Scratch wound assays at 0 h and 36 h and quantification of wound closure. (K-L) Transwell migration assays with quantification of migrated cells per field; scale bar = 100 μm. (M) Representative photographs of PANC-1 subcutaneous xenografts from the indicated groups (n = 5 per group). (N) Box plots of terminal tumor weight. (O) Tumor growth curves measured over time. Data are shown as mean ± SD; each dot represents an independent replicate. All *in vitro* experiments were performed with three independent biological replicates. Immunoblots shown are representative of three independent experiments. Statistics: one-way ANOVA test for bar box plots and two-way ANOVA for time courses. *p < 0.05, **p < 0.01, ***p < 0.001.

To elucidate the functional role of E2F3 beyond its established involvement in cell cycle regulation, we generated E2F3 knockdown and overexpression models in PANC-1 and BXPC-3 cells. Additionally, a rescue model was constructed by reintroducing exogenous E2F3 into shE2F3 cells. WB and qRT-PCR confirmed efficient modulation of E2F3 expression at both protein and mRNA levels (Figure 3A-D).

Functional assays revealed that E2F3 knockdown markedly reduced cell proliferation, while re-expression of E2F3 not only rescued proliferation but further increased growth beyond baseline levels, as measured by CCK-8 assays (Figure 3E-F). These trends were corroborated by colony formation assays, where E2F3 overexpression promoted clonogenicity, knockdown impaired colony formation, and the rescue construct restored colony forming ability (Figure 3G-H). To evaluate migratory potential, wound healing and transwell assays were performed. E2F3 overexpression enhanced cell motility, whereas knockdown suppressed migration. Re-expression of E2F3 effectively restored migratory capacity in both assays, indicating that E2F3 contributes to cellular motility (Figure 3I-L, Figure S2 A).

Although the role of E2F3 in cell-cycle regulation has been extensively characterized in multiple cancer contexts^23^, ^24^, here E2F3 refers to the E2F3A isoform, the predominant and transcriptionally active E2F3 species in PDAC cells (Figure S2G). We nevertheless reassessed its impact on cell-cycle progression in the PDAC setting. In PANC-1 cells, E2F3 overexpression promoted S-phase entry, whereas E2F3 knockdown led to G1 accumulation (Figure S2D-E). Consistently, E2F3 overexpression increased the expression of key G1/S regulators Cyclin E1 and CDK2, while E2F3 depletion led to their reduction (Figure S2F). Together, these results indicate that E2F3 retains its canonical G1/S-promoting activity in PDAC cells.

We next tested these findings *in vivo* using a subcutaneous tumorigenesis model. E2F3 knockdown significantly inhibited tumor growth, as evidenced by decreased tumor volume and slower growth kinetics (Figure 3M-O). Importantly, re-expression of E2F3 reversed this effect and further accelerated tumor progression beyond control levels. Immunofluorescence staining of harvested tumors revealed decreased Ki67 positivity in shE2F3 tumors, while rescue restored Ki67 expression, consistent with enhanced proliferative capacity (Figure S2B-C).

Collectively, these data demonstrate that E2F3 not only promotes pancreatic cancer cell proliferation and migration *in vitro* but also drives tumor growth *in vivo*, supporting its role as a pro tumorigenic factor in pancreatic cancer.

### E2F3 activates NF-κB signaling in pancreatic cancer

**Figure 4:**
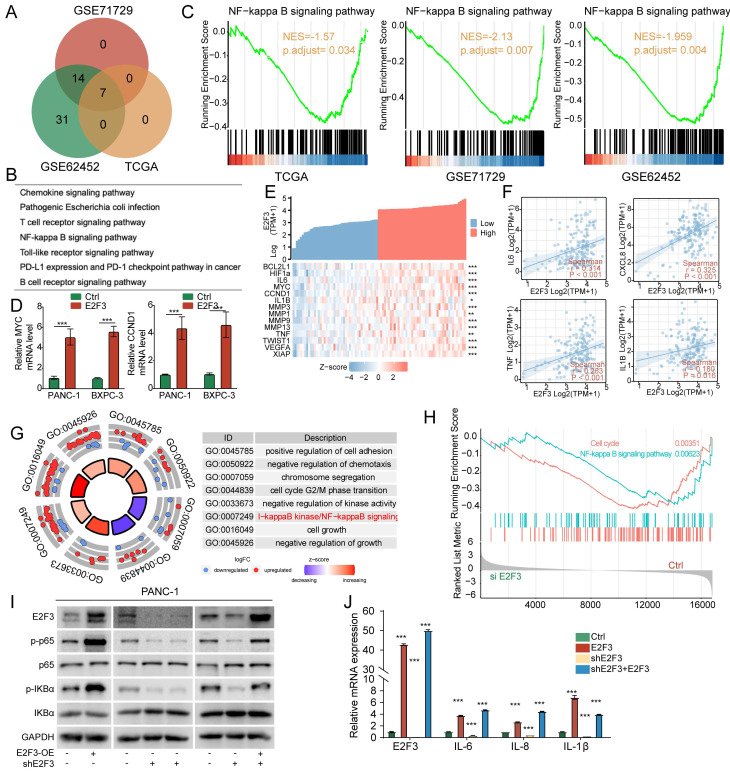
E2F3 activates the NF-κB signaling pathway in pancreatic cancer. (A) Venn diagram showing the overlap of KEGG (Kyoto Encyclopedia of Genes and Genomes) pathways enriched in high versus low E2F3 expression groups from TCGA, GSE71729, and GSE62452 cohorts, as identified by GSEA (gene set enrichment analysis). (B) Shared KEGG pathways enriched across all three datasets. (C) GSEA plots showing significant upregulation of NF-κB signaling in E2F3 high tumors across all datasets. (D) RT-qPCR analysis of NF-κB downstream targets (MYC and CCND1) after E2F3 overexpression in PANC-1 and BXPC-3 cell lines. Data represent mean ± SD from three independent biological experiments. (E) Barplot and heatmap of NF-κB target gene expression in TCGA tumors stratified by E2F3 expression. (F) Correlation analysis between E2F3 expression and representative inflammatory mediators (IL6, CXCL8, TNF, IL1B). (G) Gene Ontology Biological Process analysis of RNA-seq after E2F3 knockdown in PANC-1 cells. (H) GSEA of the RNA-seq dataset demonstrating coordinated down regulation of NF-κB and cell cycle pathway upon siE2F3-mediated silencing of E2F3. (I) Representative Western blots showing phosphorylated p65 and IκBα levels following E2F3 overexpression, knockdown, and rescue in pancreatic cancer cell lines. (J) RT-qPCR of inflammatory NF-κB target genes (IL6, CXCL8/IL8, IL1B) under the same condition. Statistical analysis was performed using Student's t-test. Data are presented as mean ± SD from three independent experiments. ***p < 0.001.

To further investigate the mechanistic basis of E2F3's impact on tumor biology, we performed gene set enrichment analysis (GSEA) across three independent PDAC cohorts (TCGA, GSE71729, and GSE62452) stratified by E2F3 expression. The intersection of enriched KEGG pathways revealed seven shared processes, with NF-κB signaling consistently upregulated in E2F3 high tumors (Figure 4A-B). GSEA enrichment plots confirmed significant activation of the NF-κB pathway in each dataset, with NES = 1.57 (TCGA, *p* = 0.034), NES = 2.13 (GSE71729, *p* = 0.007), and NES = 1.96 (GSE62452, *p* = 0.004) (Figure 4C).

To validate these associations experimentally, we measured canonical NF-κB targets. In PANC-1 and BXPC-3 cells, E2F3 overexpression markedly increased MYC and CCND1 mRNA levels (Figure 4D). Similarly, analysis of patient samples showed that NF-κB related genes, including MYC, IL6, MMPs, and VEGFA, were enriched in E2F3 high tumors, as illustrated by heatmap clustering (Figure 4E). Correlation analyses further demonstrated that E2F3 expression was positively associated with pro inflammatory cytokines IL6, CXCL8(IL8), TNF, and IL1B (Figure 4F).

To directly test whether NF-κB signaling is causally regulated by E2F3, we performed RNA-seq following E2F3 knockdown in PANC-1 cells. Building on patient derived transcriptomic associations, this *in vitro* perturbation revealed that GO biological process analysis showed suppression of multiple pathways, most notably NF-κB signaling, as well as G2/M transition and chromosome segregation, the latter consistent with E2F3's canonical cell-cycle role (Figure 4G). Concordantly, GSEA demonstrated significant downregulation of both NF-κB signaling and cell cycle programs upon E2F3 silencing (Figure 4H; Figure S3A-C).

At the protein level, immunoblotting confirmed that E2F3 overexpression enhanced phosphorylation of p65, IκBα, and upstream IKKα/β, whereas E2F3 knockdown reduced these phosphorylation events; re-expression of E2F3 restored pathway activity (Figure 4I). Consistently, RT-qPCR showed that inflammatory NF-κB targets including IL6, IL8, IL1B were transcriptionally regulated in parallel with E2F3 status (Figure 4J).

Together, these data establish E2F3 as an upstream activator of NF-κB signaling in PDAC, reinforcing its role in coupling proliferative transcriptional programs with pro inflammatory outputs.

### E2F3-driven TRIM26 promotes TAB1 K11-linked ubiquitination and canonical NF-κB activation

**Figure 5:**
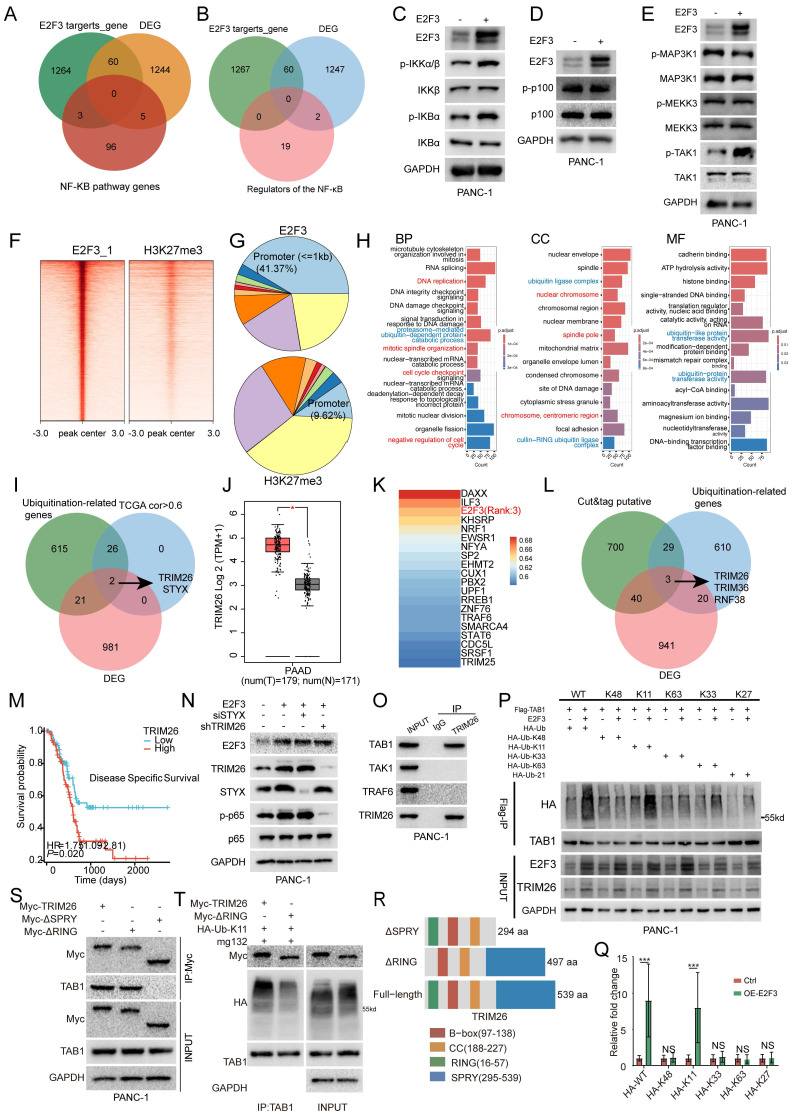
E2F3-TRIM26 drives TAB1 K11-linked ubiquitination and canonical NF-κB activation. (A) Three-way intersection of predicted E2F3 targets, E2F3 regulated DEGs (Differentially Expressed Genes), and KEGG NF-κB pathway genes shows no overlap. (B) Intersection of E2F3 targets, DEGs with known NF-κB upstream modules (IKK kinases, MAP3Ks, TRAFs, TLR adaptors) yields no candidates. (C) Immunoblots in PANC-1 cells showing that E2F3 overexpression increases phosphorylation of IKKα/β and IκBα without altering total protein, consistent with canonical NF-κB activation. (D) Non-canonical markers p100 and p-p100 are unchanged. (E) Among MAP3Ks, only TAK1 phosphorylation rises upon E2F3 overexpression, whereas total TAK1 remains stable. (F-G) E2F3 CUT&Tag (cleavage under targets and tagmentation) profiling in PANC-1 cells (n=3). Peaks are strongly promoter-proximal (41.37% within ±1 kb of TSSs), whereas H3K27me3 shows far fewer promoter peaks (9.62%), supporting assay specificity and E2F3's TF features. (H) GO analysis of genes with promoter proximal E2F3 peaks highlights classical cell cycle programs (red) and protein ubiquitination terms (blue). (I) Three set intersection to nominate E2F3 effectors that could tune TAK1 (ubiquitination genes, positive correlation with E2F3 in TCGA-PAAD, and genes suppressed by E2F3 knockdown) identifies TRIM26 and STYX. (J) TRIM26 is upregulated in tumors versus normal pancreas tissues. (K) TF ranking for TRIM26 places E2F3 among the top candidates (3rd). (L) Independent validation using an intersection of genes with E2F3 CUT&Tag promoter peaks, siE2F3 RNA-seq DEGs, and ubiquitination genes again returns a short list that includes TRIM26. (M) High TRIM26 associates with shorter disease-specific survival in PDAC (HR=1.75, p=0.020). (N) Functionally, TRIM26 but not STYX knockdown blunts E2F3 induced p-p65 activation. (O) Co-IP shows TRIM26 binds TAB1 but not TAK1 or TRAF6, indicating a TAB1-dependent route to TAK1 activation. (P) E2F3 overexpression increases total and K11-linked polyubiquitination of TAB1, with K48/K63 chains largely unchanged. (Q) Densitometric quantification of TAB1-associated ubiquitin signals from independent experiments. HA-ubiquitin intensity was normalized to immunoprecipitated TAB1.Data are presented as mean ± SD (n = 3 independent experiments). (R) Schematic of TRIM26 constructs, colors correspond to the domain legend. (S) Domain mapping: ΔSPRY (aa 295-539) fails to bind TAB1, whereas ΔRING (aa 16-57) retains binding, indicating substrate recognition via the SPRY domain. (T) Only WT-TRIM26, not ΔRING, promotes TAB1 ubiquitination, demonstrating the RING domain's requirement for E3 ligase activity. All immunoblot images are representative of three independent experiments. All quantitative experiments were performed with three independent biological replicates. Survival curves were compared using the log-rank test, and hazard ratios were estimated using Cox proportional hazards regression. Densitometric quantification was compared using Student's t-test.

To determine how E2F3 activates NF-κB, we first explored whether E2F3 transcriptionally controls core components of the pathway. Intersecting predicted E2F3 targets, E2F3-regulated DEGs, and KEGG NF-κB genes identified no overlap (Figure 5A; Supplementary Table S6). A similar intersection with known upstream regulators (IKK kinases, MAP3Ks, TRAFs, and TLR adaptors) also yielded no candidates (Figure 5B; Supplementary Table S6), arguing against direct transcriptional control of canonical NF-κB machinery.

We therefore profiled pathway activity biochemically. E2F3 overexpression increased phosphorylation of IKKα/β, IκBα without altering total protein levels, consistent with canonical NF-κB activation (Figure 5C). By contrast, p100 and p-p100 were unchanged (Figure 5D), excluding noncanonical signaling. Among MAP3Ks that feed into IKKs, only TAK1 phosphorylation was selectively elevated by E2F3 while total TAK1 was stable (Figure 5E), positioning TAK1 as a proximal effector of E2F3.

To nominate effectors that could account for TAK1 activation, we performed CUT&Tag profiling for E2F3 in PANC-1 cells (n=3). E2F3 peaks were strongly promoter proximal (41.37% within ±1 kb of TSSs), whereas the control mark H3K27me3 showed far fewer promoter peaks (9.62%), supporting assay specificity and E2F3's TF behavior (Figure 5F-G; Figure S4A-B; Supplementary Table S7). GO analysis of genes bearing E2F3 promoter peaks highlighted classical cell cycle programs and, notably, terms related to protein ubiquitination, suggesting that E2F3 may activate NF-κB by upregulating ubiquitin pathway components that tune TAK1 (Figure 5H).

Because TAK1 output is governed by polyubiquitination, particularly K63 and K11 linkages on TAB1-TAB3, we intersected three datasets to find candidate E2F3 effectors: a curated ubiquitination gene list, genes positively correlated with E2F3 in TCGA-PAAD (Pearson R > 0.6), and genes downregulated upon E2F3 knockdown. Two genes emerged, TRIM26 and STYX (Figure 5I). TRIM26, but not STYX, was upregulated in tumors versus Normal tissue (Figure 5J; Figure S4C) and correlated strongly with E2F3 expression (R=0.67; Figure S4D-E). Ranking transcription factors associated with TRIM26 placed E2F3 among the top candidates (3rd; Figure 5K), whereas no such relationship was observed for STYX (Figure S4F, rank54), indicating a more specific E2F3-TRIM26 link.

We validated this prioritization using an independent strategy: intersecting genes with promoter proximal E2F3 CUT&Tag peaks, siE2F3 RNA-seq DEGs (Supplementary Table S8), and ubiquitination genes. This again yielded a short list that included TRIM26 supporting the robustness of our screen (Figure 5L), Clinically, high TRIM26 expression associated with shorter disease specific survival in PDAC (HR = 1.75, *p* = 0.020; Figure 5M), and showed a trend toward worse overall survival that did not reach statistical significance (Figure S4G). STYX expression was negatively correlated with both disease specific and overall survival, indicating an adverse prognostic impact (Figure S4H-I). Functionally, only TRIM26 knockdown attenuated E2F3-induced p-p65 activation, whereas STYX depletion had no detectable effect on NF-κB signaling (Figure 5N), indicating that TRIM26, but not STYX, functionally mediates the E2F3-TAK1/NF-κB axis in PDAC cells. Although STYX also showed prognostic association in PDAC, these results suggest that its contribution to tumor progression may involve oncogenic signaling pathways distinct from NF-κB.

To delineate the mechanism, we mapped TRIM26 interactions within the pathway. Co-immunoprecipitation revealed selective binding of TRIM26 to TAB1, but not to TAK1 or TRAF6, indicating a TAB1 dependent route to TAK1 activation (Figure 5O). Consistent with this, E2F3 overexpression increased total and K11 linked polyubiquitination of TAB1, whereas K48 and K63 linked chains were largely unchanged, pointing to a specific, non-degradative K11 signal on TAB1 (Figure 5P-Q). In addition, using an E3-inactive TRIM26 mutant (ΔRING), we found that wild-type TRIM26, but not ΔRING, enhanced TAB1-TAK1 association and increased TAK1 phosphorylation (Thr187), supporting a functional coupling between TAB1 K11-linked ubiquitination and TAK1 activation (Figure S5A).

Domain mapping showed how TRIM26 executes this function. We generated two TRIM26 truncations: ΔRING (aa 16-57), and ΔSPRY (aa 295-539) (Figure 5R). ΔSPRY failed to bind TAB1, whereas ΔRING retained binding (Figure 5S), indicating that the SPRY domain mediates substrate recognition. However, only wild type TRIM26 not the ΔRING mutant promoted TAB1 ubiquitination despite comparable binding (Figure 5T), demonstrating that the RING domain is indispensable for E3 ligase activity.

Together, these data define a coherent cascade: E2F3 transcriptionally upregulates TRIM26, TRIM26 engages TAB1 via its SPRY domain and installs K11-linked polyubiquitin through its RING domain, K11-modified TAB1 stabilizes the TAB1-TAK1 complex and augments TAK1 phosphorylation, thereby driving canonical NF-κB activation in pancreatic cancer cells.

### E2F3 directly activates TRIM26 transcription by binding to its promoter

**Figure 6:**
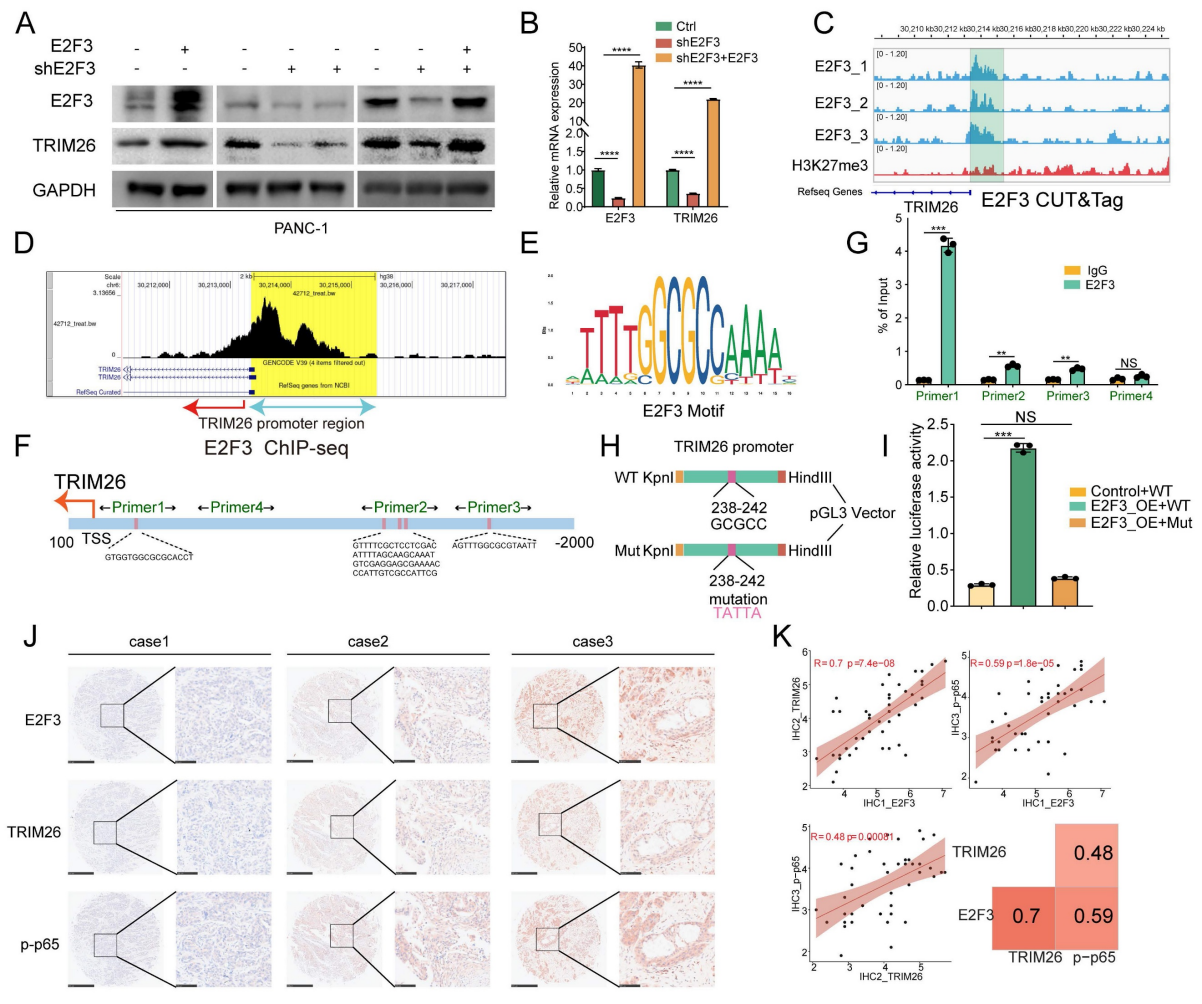
E2F3 directly binds the TRIM26 promoter and drives its transcription in PDAC. (A-B) Rescue experiments in E2F3-silenced PDAC cells: Representative immunoblots and qRT-PCR analysis show that re-expression of E2F3 restores TRIM26 at protein (WB) and mRNA (qRT-PCR) levels. (C) E2F3 CUT&Tag tracks (three biological replicates) show strong enrichment at the TRIM26 promoter area; H3K27me3 lacks focal enrichment. (D) Public E2F3 ChIP-seq (GSM1239452) confirms a prominent peak at the TRIM26 promoter. (E-F) JASPAR motif analysis identifies six putative E2F3 motifs within -2000 to +100 bp of the TRIM26 TSS, grouped into three clusters. (G) ChIP-qPCR with four primer sets demonstrates most significant E2F3 occupancy at the Primer1 region; this region overlaps the CUT&Tag peak located approximately 182-387 bp upstream of the TSS. (H)Schematic of luciferase reporter constructs containing the TRIM26 promoter (-400 to +100 bp relative to TSS, WT or mutant). The E2F3 core motif (GCGCC, -238 to -242 bp) was mutated to TATTA in the mutant construct. (I) Dual luciferase reporter assays using the TRIM26 promoter (-400 to +100 bp). Co-expression of E2F3 increases Firefly/Renilla activity versus vector; mutation of the core E2F motif (GCGCC-TATTA within the Primer1 region) nearly abolishes activation. (J) Representative IHC images of E2F3, TRIM26, and p-p65 in PDAC tissue microarrays, Scale bars, 500 µm (low magnification) and 100 µm (high magnification). (K) Correlation analysis of IHC scores showing strong positive associations: E2F3-TRIM26 (R = 0.70, p = 7.4e-08), E2F3-p-p65 (R = 0.59, p = 1.8e-05), and TRIM26-p-p65 (R = 0.48, p = 0.00081). Heatmap summary of correlation coefficients is shown at right. All quantitative data are presented as mean ± SD from three independent experiments. Statistical significance was assessed using Student's t-test for qRT-PCR, ChIP-qPCR, and luciferase assays, and Spearman's rank correlation for IHC analyses. *** p < 0.001; NS, not significant.

To validate that TRIM26 is a downstream transcriptional target of E2F3, we performed rescue experiments in E2F3 silenced PDAC cells. Re-expression of E2F3 restored TRIM26 mRNA and protein levels, as confirmed by qRT-PCR and western blot (Figure 6A-B).

We next assessed whether E2F3 directly binds to the TRIM26 promoter. CUT&Tag profiling in PANC-1 cells (three replicates) revealed strong enrichment of E2F3 peaks near the TRIM26 TSS, whereas the repressive histone mark H3K27me3 showed diffuse distribution without focal enrichment (Figure 6C). Publicly available ChIP-seq data (GSM1239452) further corroborated the presence of a prominent E2F3 binding peak at the TRIM26 promoter (Figure 6D). Together, these findings support that TRIM26 is a direct transcriptional target of E2F3.

Motif analysis using the JASPAR database identified six putative E2F3 binding motifs within the -2000 to +100 bp region of the TRIM26 promoter, clustered into three regions (Figure 6E-F, Table S4). ChIP-qPCR using four primer sets spanning these regions detected E2F3 enrichment across multiple sites, with the strongest occupancy observed at the region amplified by Primer 1 (Figure 6G). Notably, this binding region overlapped with the CUT&Tag peak localized 182-387 bp upstream of the TSS.

To directly test whether E2F3 activates TRIM26 transcription, we cloned the TRIM26 promoter (-400 to +100 bp relative to the TSS) into the pGL3-Basic luciferase vector (Figure 6H). Dual-luciferase assays demonstrated that co-expression of E2F3 significantly increased Firefly activity relative to Renilla control compared with vector control (Figure 6I). Importantly, site directed mutation of the core E2F3 motif (GCGCC at -238 to -242 bp, mutated to TATTA) within the Primer 1(Figure 6H) enriched region nearly abolished E2F3 induced promoter activation, as indicated by reduced Firefly/Renilla activity compared with the wild type construct (Figure 6I). These results confirm that an intact E2F3 binding motif is essential for TRIM26 transcriptional activation.

Finally, to evaluate clinical relevance, immunohistochemistry staining of PDAC tissue microarrays demonstrated positive correlations between E2F3 and TRIM26, as well as between E2F3 and phosphorylated p65 (p-p65) (Figure 6J). Quantitative correlation analysis further supported these relationships: E2F3-TRIM26 (R = 0.70, p = 7.4e-08), E2F3-p-p65 (R = 0.59, p = 1.8e-05), and TRIM26-p-p65 (R = 0.48, p = 0.00081) (Figure 6K). Collectively, these results demonstrate that E2F3 directly binds and transcriptionally activates TRIM26, thereby driving canonical NF-κB signaling in pancreatic cancer.

### TRIM26 transmits E2F3 signaling and is targetable in PDAC

**Figure 7:**
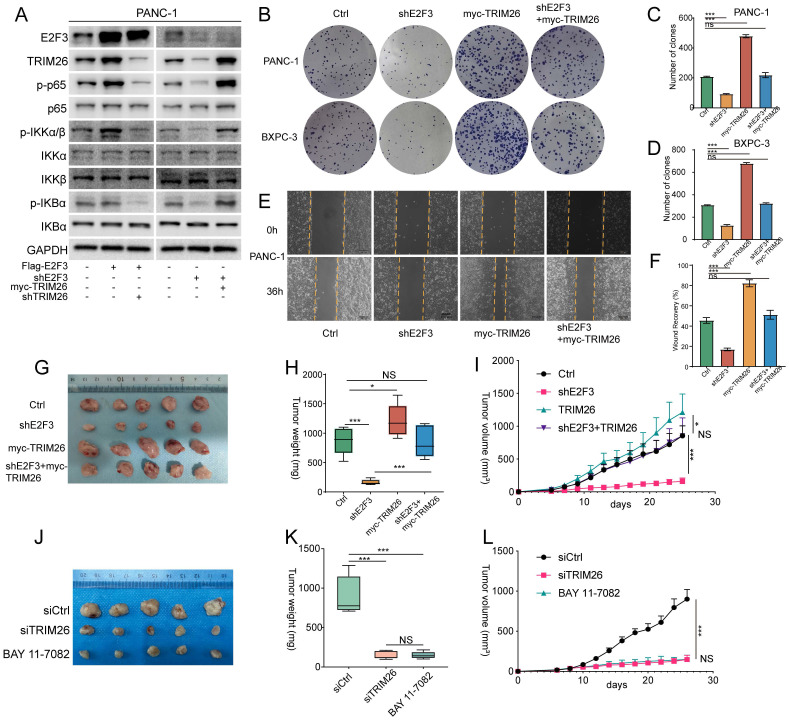
TRIM26 links E2F3 to NF-κB and is therapeutically tractable in PDAC xenografts. (A) Immunoblots in PDAC cells showing that TRIM26 is required for E2F3 driven pathway activation. (B) colony formation assays (PANC-1, BxPC-3) for the indicated conditions. (C-D) Quantification of colonies in (B). Bars represent mean ± SD (n=3 independent experiments). (E) Wound healing assays at 0 h and 36 h; dashed lines mark initial wound edges. Scale bar, 100 μm. (F) Quantification of relative wound closure for (E) (mean ± SD, n=3). (G) Representative tumors from xenograft cohorts (Ctrl, shE2F3, myc-TRIM26, shE2F3+myc-TRIM26) (n = 5 per group). (H) Tumor weights at experimental endpoint for cohorts in (G). Data are presented as mean ± SD (n = 5 per group). (I) Tumor growth curves for cohorts in (G) measured every 2 days. Mean ± SD (n = 5 per group). (J) Representative tumors from therapeutic groups (siControl, siTRIM26, BAY 11-7082, n = 5 per group). (K) Endpoint tumor weights for groups in (J). Mean ± SD (n = 5 per group). (L) Tumor growth curves for groups in (J) showing siTRIM26 efficacy comparable to BAY 11-7082. Mean ± SD (n = 5 per group). Statistical analysis was performed using one-way ANOVA with Tukey's post hoc test for multi-group comparisons or two-tailed Student's t-test where appropriate. *P < 0.05, *** P < 0.001; ns: not significant.

To further establish the functional relevance of the E2F3-TRIM26-NF-κB axis, we performed reciprocal rescue experiments. In PDAC cells, TRIM26 knockdown markedly attenuated the E2F3 induced phosphorylation of p65, indicating that TRIM26 is required for E2F3 driven NF-κB activation. Conversely, TRIM26 overexpression restored NF-κB signaling in E2F3-silenced cells, as reflected by recovered p-p65 levels (Figure 7A). Phenotypically, TRIM26 overexpression partially reversed the reduction in colony forming capacity caused by E2F3 silencing (Figure 7B-D), and yielded similar rescue effects in wound healing assays of cell migration (Figure 7E-F). To further confirm that the oncogenic effects of E2F3 are functionally dependent on NF-κB signaling, pharmacologic inhibition of NF-κB pathway using BAY 11-7082 markedly attenuated E2F3-induced proliferation and migration in PDAC cells (Figure S6A-D), supporting a critical role of NF-κB as the downstream effector of the E2F3-TRIM26 axis.

*In vivo*, xenograft models further supported these findings. E2F3 silencing significantly impaired tumor growth, whereas TRIM26 overexpression promoted tumor progression. Notably, overexpression of TRIM26 in E2F3 silenced tumors restored growth to levels comparable with controls (Figure 7G-I). These results suggest that the survival benefit derived from E2F3 inhibition is largely attributable to suppression of TRIM26 and downstream NF-κB signaling, rather than solely from E2F3 dependent cell cycle control, which may be buffered by redundant mechanisms. Consistent with this interpretation, immunoblot analysis of xenograft tumors further showed that E2F3 silencing reduced TRIM26 expression and NF-κB pathway activation, as reflected by decreased p-p65 and p-TAK1 levels, whereas TRIM26 re-expression partially restored these signals *in vivo* (Supplementary Figure S6E).

Finally, we explored the translational potential of targeting TRIM26. Given the broad physiological functions of NF-κB and the toxicity that has limited the clinical application of systemic NF-κB inhibitors^25^ and considering the inherent challenges in directly targeting transcription factors^26^, we reasoned that TRIM26 may represent a more feasible therapeutic node within this axis. To test this, we designed TRIM26 specific siRNAs, chemically stabilized siRNAs (5′-cholesterol conjugated, fully 2′-O-methyl modified) were prepared at 1 mg/mL and administered at 1 mg/kg twice weekly. Treatment with siTRIM26 significantly suppressed tumor growth, resulting in slower growth kinetics and reduced final tumor volume compared with the vehicle control. (Figure 7J-L, Figure S6F-G). The magnitude of inhibition was comparable to BAY 11-7082, indicating that TRIM26 knockdown is sufficient to phenocopy pharmacologic blockade of the pathway (Figure 7K). Mechanistically, tumors from the siTRIM26 group showed decreased p-p65 and reduced expression of canonical NF-κB target genes, consistent with on target pathway inhibition.

**Figure 8:**
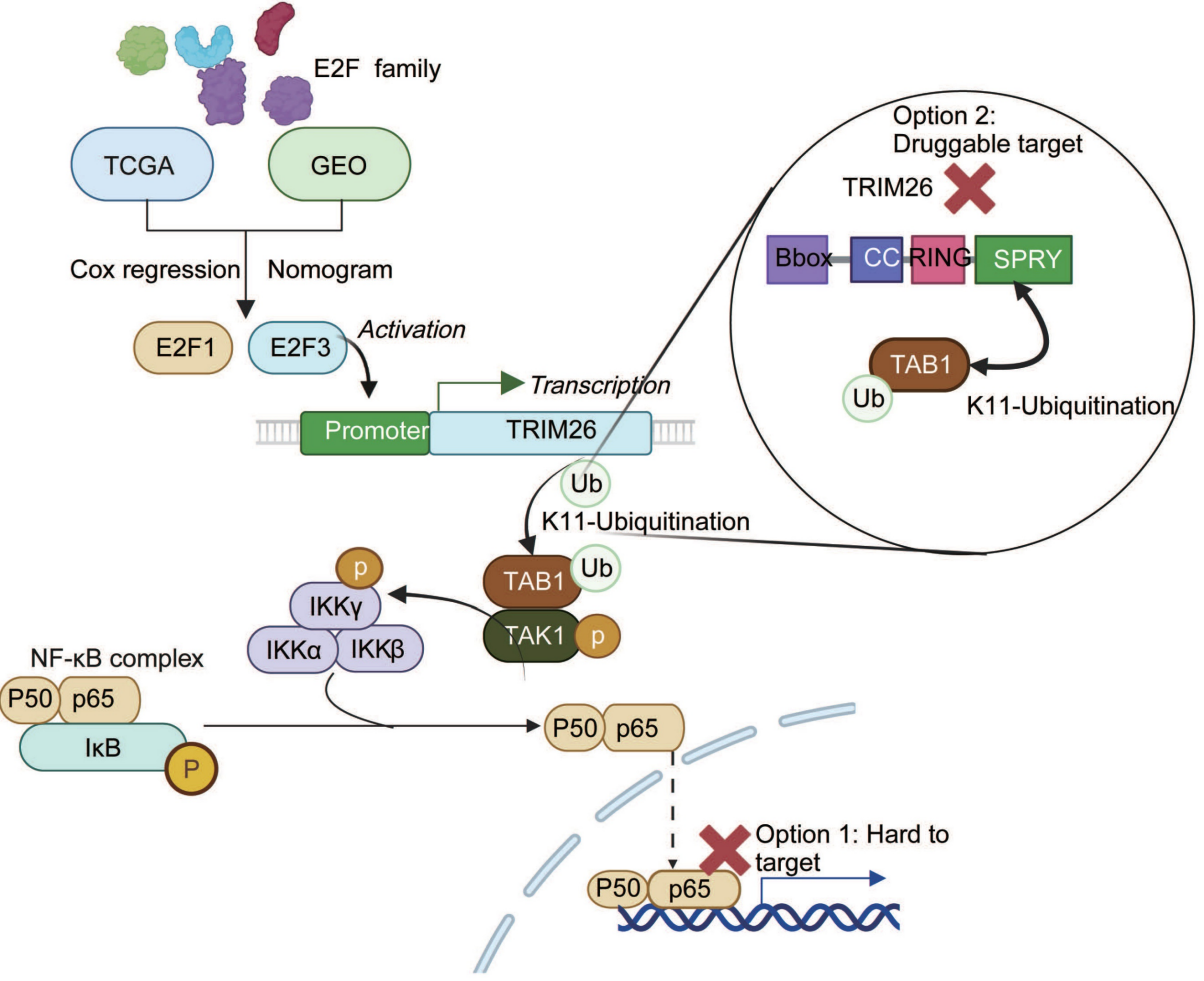
Mechanistic model of the E2F3-TRIM26-TAB1-NF-κB axis in PDAC. E2F3 transcriptionally upregulates TRIM26, which catalyzes K11-linked ubiquitination of TAB1, stabilizing the TAB1-TAK1 complex and activating canonical NF-κB signaling. Direct inhibition of NF-κB (p65) is difficult due to systemic toxicity, whereas targeting TRIM26, a druggable E3 ligase upstream of TAK1, offers a more feasible therapeutic strategy.

Collectively, our study identifies E2F3 as a key oncogenic driver in PDAC that promotes NF-κB activation through TRIM26 mediated TAB1 ubiquitination, thereby accelerating tumor progression (Figure 8). These findings not only provide mechanistic insights into the crosstalk between E2F and NF-κB pathways but also propose TRIM26 as a potential therapeutic target for translational intervention.

## Discussion

This study establishes a clinically anchored, mechanistic link between the E2F program and NF-κB signaling in PDAC. Starting from a family wide screen across TCGA-PAAD and independent cohorts, we prioritized E2F3 as a prognostically adverse factor with an impact comparable to E2F1, validated E2F3 overexpression in tumors, and demonstrated that E2F3 drives PDAC growth *in vitro* and *in vivo*. Mechanistically, E2F3 directly upregulates TRIM26, which installs K11-linked polyubiquitin on TAB1, stabilizes the TAB1-TAK1 complex, and activates canonical NF-κB. Bidirectional rescue at the signaling and phenotypic levels, together with positive correlations among E2F3, TRIM26, and p-p65 in patient tissues, supports the functional relevance of this axis.

E2F3 encodes two isoforms, E2F3A and E2F3B, which arise from alternative promoters and exert distinct biological functions. E2F3A peaks at the G1/S transition and is tightly coupled to proliferative programs and tumorigenesis, whereas E2F3B is preferentially expressed in quiescent cells and associated with homeostatic regulation^27^. Most clinical datasets and commonly used antibodies do not distinguish between these two isoforms. Given that proliferative drive is a central feature of tumor biology, we focused on E2F3A in our functional studies. Using the full length E2F3A transcript, we observed that its expression was sufficient to rescue proliferative phenotypes and restore NF-κB activation in PDAC cells, thereby supporting E2F3A as the predominant oncogenic isoform operative in this context.

The biological context of PDAC provides a rationale for the emergence of E2F3 as a key oncogenic node. Widespread RB pathway deregulation, most commonly via CDKN2A/p16 loss, together with near-universal KRAS activation, creates a sustained dependence on G1/S transcriptional output and E2F activity^28^. Consistent with this model, the adverse prognostic impact of E2F3 was most evident in early-stage disease and attenuated in advanced tumors, where additional oncogenic programs and microenvironmental constraints may dominate^29^. These findings suggest that E2F3 and TRIM26 may serve as biomarkers for risk stratification and as candidates for adjuvant intervention following resection.

TRIM26 has been reported to exert divergent functions across tumor types. In hepatocellular carcinoma, TRIM26 promotes tumor progression by preserving cell survival and by engaging proliferative programs such as Wnt/β-catenin^30^. In colorectal cancer, elevated TRIM26 correlates with poorer outcomes, consistent with a pro inflammatory, NF-κB competent state^31^, ^32^. By contrast, TRIM26 can be tumor suppressive in certain settings. In clear cell renal cell carcinoma it promotes ETK degradation to dampen AKT/mTOR^33^, and in osteosarcoma it destabilizes RACK1^34^, together highlighting a context dependent role for TRIM26. Our data argue that in PDAC, TRIM26 functions as a pro-tumorigenic factor by catalyzing K11 linked TAB1 ubiquitination to stabilize the TAB1-TAK1 complex and drive canonical NF-κB activation. Although TRIM family E3 ligases often regulate multiple substrates, both prior evidence^35^ and our data support TAB1 as the principal mediator linking TRIM26 to TAK1 activation in this context, whereas TAB2 and TAB3 mainly function as ubiquitin-binding adaptors within the TAK1 complex.

Although E2F3 is classically linked to cell cycle control, we did not reemphasize this established role. Instead, we asked whether E2F3 couples proliferation with inflammatory survival cues. Transcriptomic analyses consistently connected E2F3 to NF-κB activation, and biochemical assays localized the effect to the canonical TAK1-IKK-IκBα-p65 cascade. TAK1 is activated through multiple inputs, including TAB2/3-mediated recognition of K63 linked ubiquitin chains^36^, ^37^ and TAB1 dependent allosteric mechanisms^38^, ^39^. Our data support a complementary route: TRIM26 binds TAB1 via its SPRY domain and, in a RING-dependent manner, increases K11-linked ubiquitin on TAB1, thereby enhancing TAK1 phosphorylation. The absence of detectable TRIM26-TAK1 co-immunoprecipitation is consistent with a model in which TRIM26 modifies the scaffold (TAB1) rather than the kinase directly, promoting complex assembly without a stable TRIM26-TAK1 interface. While we did not map TAB1 acceptor lysines, the selective increase of K11 chains on TAB1, the requirement for the TRIM26 RING domain, and our domain mapping data together support this model and motivate site resolved validation.

Although STYX was associated with poor prognosis in PDAC, it did not mediate E2F3-dependent NF-κB activation in our assays, despite its reported role as a catalytically inactive DUSP family member involved in MAPK regulation^40^. In contrast, TRIM26 emerged as the primary mediator linking E2F3 to the TAK1-IKK-p65 axis. Functionally, TRIM26 was indispensable for E2F3-driven oncogenic signaling both *in vitro* and *in vivo*, suggesting that the therapeutic benefit of E2F3 inhibition primarily reflects suppression of the E2F3-TRIM26-NF-κB pathway rather than cell-cycle outputs buffered by redundancy among E2F paralogs.

Recent advances in RNAi therapeutics have renewed the translational potential of gene silencing strategies. The landmark FDA approval of Patisiran in 2018 for hereditary transthyretin amyloidosis established RNAi as a clinically viable modality^41^. In oncology, continued improvements in nucleic acid delivery such as lipid nanoparticles, polymeric carriers, and bio derived vesicles^42^, ^43^ have markedly expanded the feasibility of RNA based interventions. In this study, we employed chemically stabilized siRNAs (5′-cholesterol conjugated, fully 2′-O-methyl modified) and showed that systemic administration of siTRIM26 significantly suppressed PDAC tumor growth *in vivo*. Importantly, this treatment achieved an inhibitory effect comparable to pharmacologic NF-κB blockade. These findings provide proof of concept that targeting TRIM26 through RNAi offers a tractable and safer route to attenuate the E2F3-TRIM26-NF-κB axis in pancreatic cancer. We note that BAY 11-7082, although widely used as an NF-κB pathway inhibitor, is not fully specific and has reported off-target activities, including effects on certain deubiquitinases; therefore, the BAY 11-7082 data should be interpreted as supportive pharmacologic evidence rather than as the sole basis for mechanistic inference. Collectively, our results highlight TRIM26 as a druggable node within a pathway otherwise difficult to target, given that E2F3 is a transcription factor and systemic NF-κB blockade is poorly tolerated. The enzymatic RING E3 ligase activity of TRIM26 provides entry points for small molecule inhibitors, ligand directed degraders, or molecular glues, while siRNA-based silencing offers an orthogonal nucleic acid therapeutic strategy.

## Conclusion

In summary, this study identifies E2F3 as a key oncogenic transcription factor in PDAC that promotes tumor progression through transcriptional upregulation of TRIM26. Mechanistically, TRIM26 catalyzes K11-linked ubiquitination of TAB1, thereby activating canonical NF-κB signaling. Functionally, the E2F3-TRIM26 axis supports tumor growth both *in vitro* and *in vivo*, and TRIM26 inhibition attenuates these effects. Importantly, targeting TRIM26 using chemically stabilized siRNAs produced tumor suppression comparable to pharmacologic NF-κB blockade, suggesting a potential route to modulate this pathway with reduced systemic toxicity. These findings provide mechanistic insight into the connection between E2F driven transcription and inflammatory signaling in PDAC and highlight TRIM26 as a tractable target for future therapeutic exploration.

## Supplementary Material

Supplementary figures and tables 1-5.

Supplementary table 6: Intersection of E2F3 targets, DEGs, and NF-κB-related genes.

Supplementary table 7: E2F3 CUT&Tag peak annotation.

Supplementary table 8: DEGs upon E2F3 silencing.

## Figures and Tables

**Table 1:** Univariate and multivariate Cox regression analyses of E2F family members and clinical parameters in TCGA-PAAD cohort

Characteristics	Comparison	Univariate HR (95% CI)	P value	Multivariate HR (95% CI)	P value
E2F1	High vs. Low	1.737 (1.146-2.634)	0.009	2.002 (1.082-3.703)	0.027
E2F2	High vs. Low	1.474 (0.974-2.229)	0.066	0.800 (0.455-1.406)	0.438
E2F3	High vs. Low	1.428 (0.942-2.165)	0.093	1.347 (0.867-2.093)	0.185
E2F4	High vs. Low	1.130 (0.749-1.704)	0.561	-	-
E2F5	High vs. Low	1.034 (0.686-1.558)	0.873	-	-
E2F6	High vs. Low	0.779 (0.513-1.182)	0.241	-	-
E2F7	High vs. Low	1.545 (1.018-2.344)	0.041	0.862 (0.518-1.433)	0.566
E2F8	High vs. Low	1.379 (0.911-2.086)	0.129	-	-
T stage	T3&T4 vs. T1&T2	2.023 (1.072-3.816)	0.030	2.009 (1.022-3.950)	0.043
Age	>65 vs ≤65	1.290 (0.854-1.948)	0.227	-	-
Gender	Male vs Female	0.809 (0.537-1.219)	0.311	-	-
Histologic grade	G3&G4 vs G1&G2	1.538 (0.996-2.376)	0.052	1.311 (0.838-2.050)	0.235

## Data Availability

All data supporting the findings of this study are included in the manuscript and its supplementary materials. The raw sequencing datasets generated during this study have been deposited in the Genome Sequence Archive (GSA) at the National Genomics Data Center under BioProject accession PRJCA057559 and will be released upon publication.

## Abbreviations

CCK-8: Cell Counting Kit-8
ChIP: chromatin immunoprecipitation
CUT&Tag: Cleavage Under Targets and Tagmentation
DSS: disease-specific survival
GEO: Gene Expression Omnibus
GO: Gene Ontology
GSEA: gene set enrichment analysis
HR: hazard ratio
IHC: immunohistochemistry
IKK: IκB kinase
IP: immunoprecipitation
KEGG: Kyoto Encyclopedia of Genes and Genomes
NF-κB: nuclear factor kappa B
OE: overexpression
PAAD: pancreatic adenocarcinoma
PDAC: pancreatic ductal adenocarcinoma
PFI: progression free interval
RT-qPCR: quantitative reverse transcription PCR
RNA-seq: RNA sequencing
shRNA: short hairpin RNA
SPF: specific pathogen-free
WB: western blot
ANT: adjacent non-tumor tissue
N: normal pancreatic tissue
T: pancreatic tumor tissue
DEGs: Differentially Expressed Genes
