# Supplementary material for: E2F3 activates NF-κB signaling through TRIM26 mediated TAB1 ubiquitination in pancreatic cancer
Source: Int J Biol Sci. 2026 Apr 8;22(8):4243–61. doi: 10.7150/ijbs.127710 (PMC13137955; doi:10.7150/ijbs.127710)
Supplement: Supplementary file 1 — Supplementary figures and tables 1-5. [file ijbsv22p4243s1.pdf]

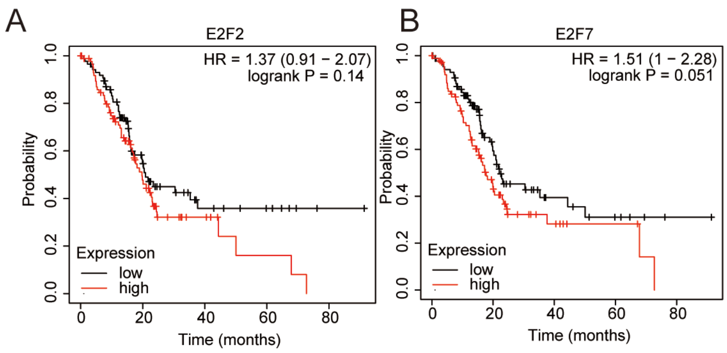

Figure S1. Kaplan–Meier survival analyses of PDAC patients stratified by median expression of E2F2 and E2F7.(A) Kaplan–Meier survival curves showing overall survival stratified by E2F2 expression levels (high vs. low).(B) Kaplan–Meier survival curves showing overall survival stratified by E2F7 expression levels (high vs. low). Statistical analysis was performed using the log-rank test.

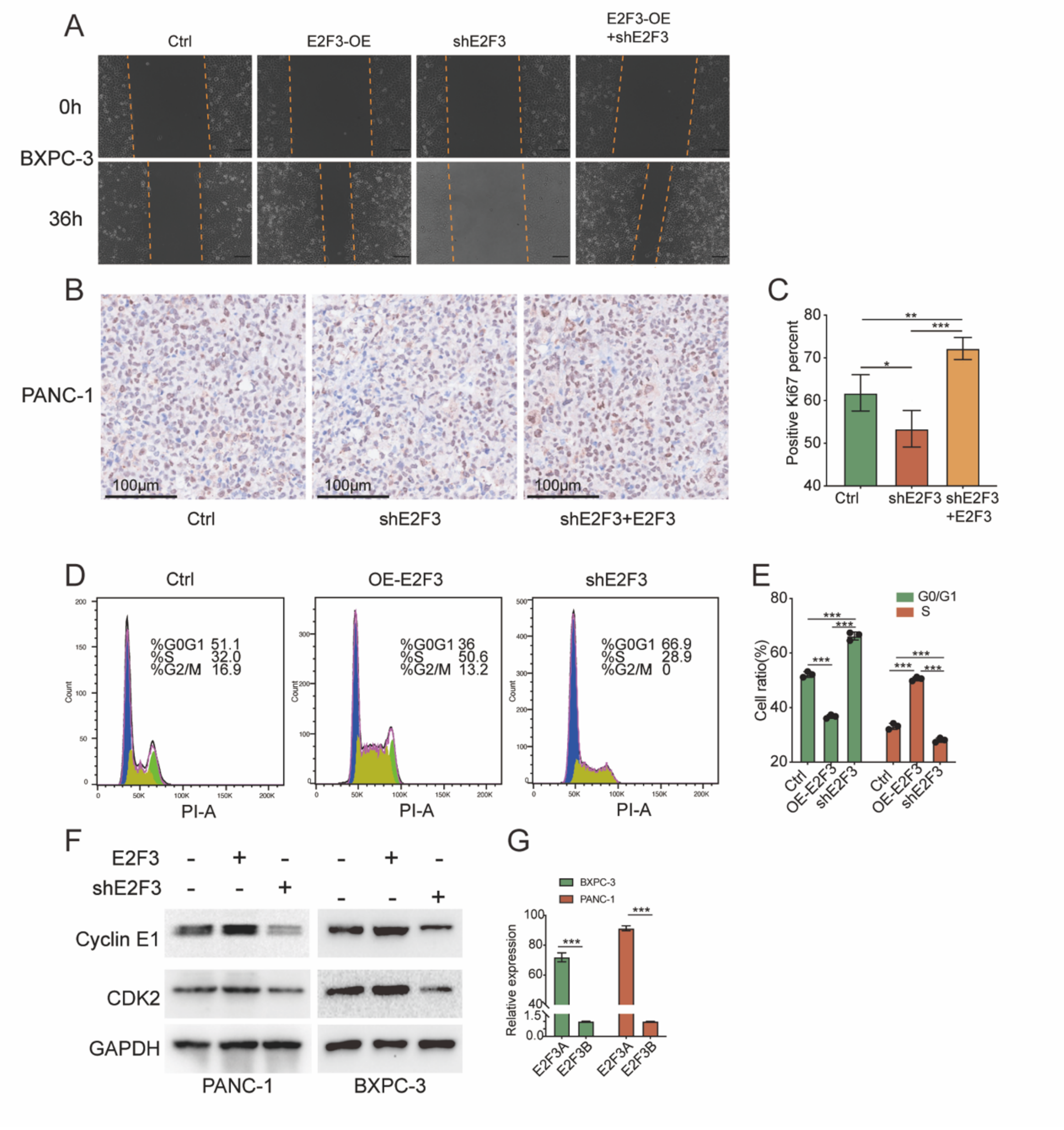

Figure S2. Additional functional and histological validation of E2F3 activity in PDAC. (A)Representative wound-healing images of BxPC-3 cells from control, shE2F3, and shE2F3 + E2F3 rescue groups. Scale bars, 100 µm. (B) Representative immunofluorescence images of Ki67 staining in xenograft tumors from the same groups, showing reduced Ki67 positivity after E2F3 knockdown and marked increase upon rescue. Scale bars, 100 µm. (C) Quantification of Ki67-positive cells across groups. (D) Flow cytometric analysis of cell-cycle distribution in PANC-1 cells following E2F3 overexpression or knockdown, assessed by PI staining.(E) Quantification of the percentages of cells in G0/G1 and S phases from (D). Data are presented as mean ± SD. (F) Immunoblot analysis of Cyclin E1 and CDK2 protein levels in PANC-1/BXPC-3 cells with E2F3 overexpression or knockdown, with GAPDH serving as a loading control. (G) Quantification reveals that E2F3A is the predominant isoform, whereas E2F3B is expressed at markedly lower levels. Data are mean ± SD. Statistical analysis was performed using Student's t-test.*p < 0.05, **p < 0.01, ***p < 0.001.

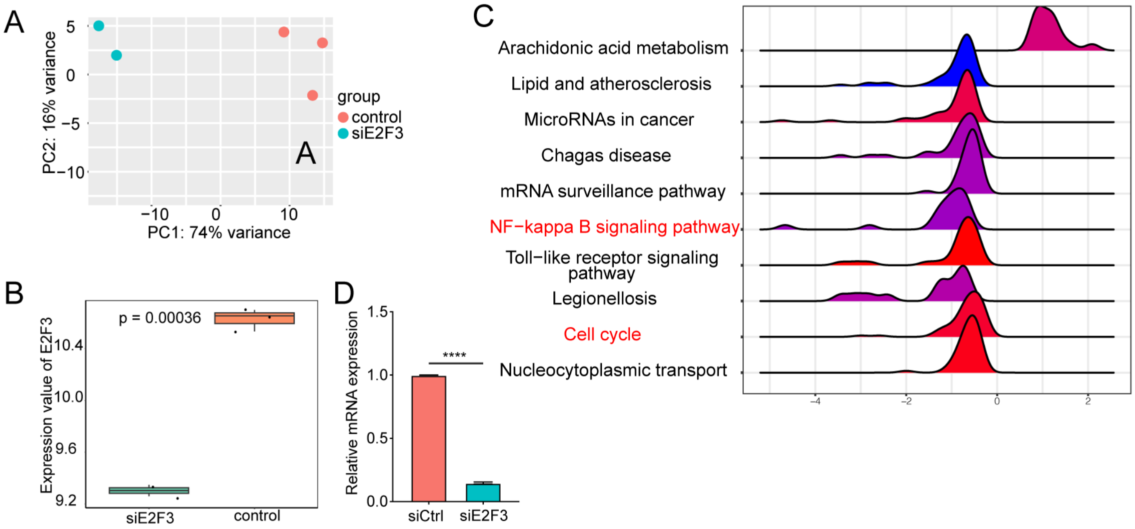

Figure S3. Transcriptomic profiling of E2F3 knockdown in PANC-1 cells. (A) Principal component analysis (PCA) of bulk RNA-seq data from control and siE2F3 PANC-1 cells, showing clear separation between groups. (B) Boxplot of RNA-seq data validating effective knockdown of E2F3, with significantly decreased E2F3 mRNA levels in the siE2F3 group compared to control. (C) Ridge plot of GSEA results depicting the top 10 significantly enriched pathways following E2F3 silencing. Notably, NF-κB signaling and cell cycle related pathways were prominently represented among these changes. (D) Validation of siE2F3 knockdown efficiency by qRT-PCR. Statistical analysis was performed using Student's *t*-test. Data are presented as mean ± SD. ****$p < 0.0001$.

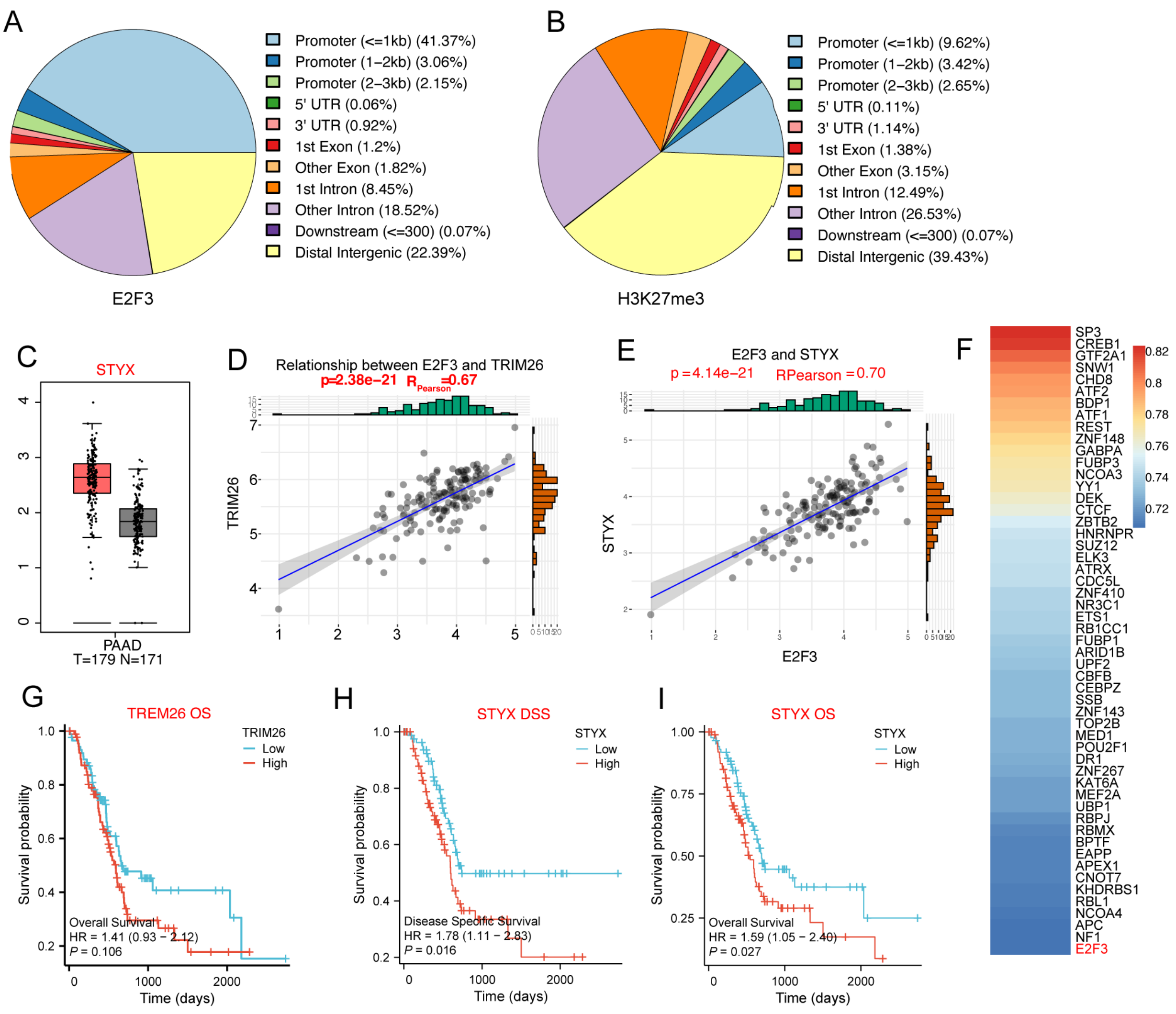

Figure S4. Extended analyses of E2F3 associated ubiquitination genes in PDAC.(A-B) Genome wide CUT&Tag profiling of E2F3 in PANC-1 cells (n = 3) showing genomic peak distribution compared with H3K27me3 control. Promoters were defined as ±1 kb around annotated TSSs, showing strong promoter bias of E2F3 peaks (41.37%) versus H3K27me3 (9.62%), confirming TF-like binding behavior and assay specificity. (C) STYX mRNA expression levels in PDAC tumors compared with normal pancreatic tissue. (D) Correlation analysis of TRIM26 expression with E2F3 expression in TCGA-PAAD transcriptomes, revealing a strong positive association. (E) Correlation analysis of STYX expression with E2F3 expression in TCGA-PAAD transcriptomes, showing positive association. (F) Transcription factor regulon ranking associated with STYX expression. Unlike TRIM26, E2F3 is not among the top regulators for STYX (ranked 54). (G) Overall survival analysis of TRIM26 expression in PDAC, showing a trend toward worse prognosis that did not reach statistical significance. (H) Disease-specific survival analysis of STYX expression in PDAC, showing an adverse prognostic association. (I) Overall survival analysis of STYX expression in PDAC, confirming poor prognostic correlation.Survival curves were compared using the log-rank test, and hazard ratios were estimated using Cox proportional hazards regression.

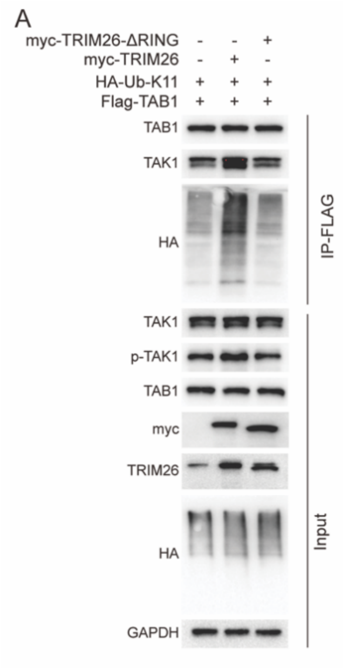

Figure S5A. TRIM26-mediated K11-linked ubiquitination of TAB1 promotes TAB1–TAK1 complex assembly and TAK1 activation.

PANC-1 cells were co-transfected with Flag-TAB1 and HA-Ub(K11) together with either wild-type TRIM26 or an E3 ligase-inactive ΔRING mutant. Flag-TAB1 was immunoprecipitated using anti-Flag antibodies and immunoblotted for HA to assess K11-linked ubiquitination and for TAK1 to evaluate TAB1–TAK1 complex formation.

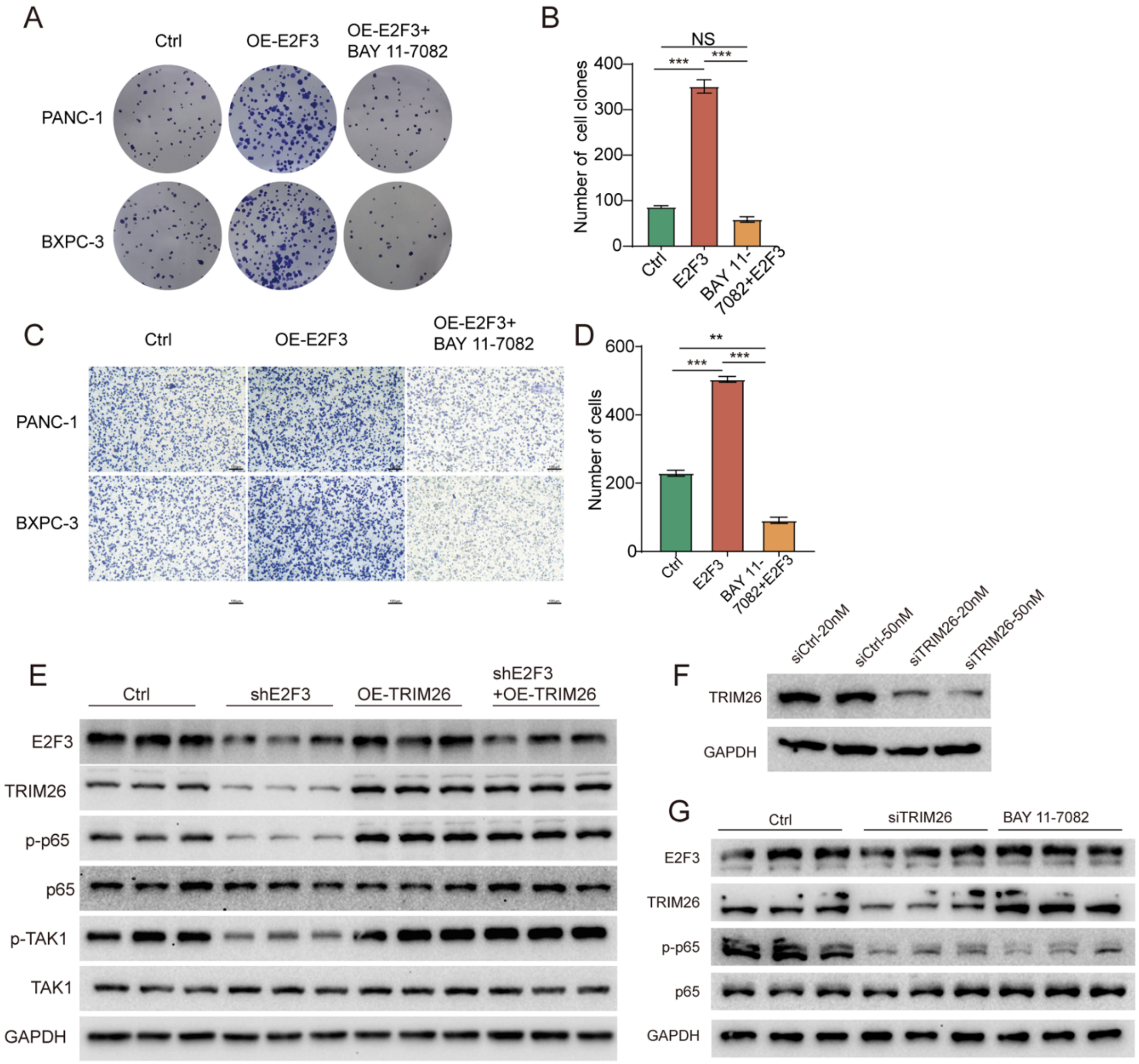

Figure S6.NF-κB inhibition attenuates E2F3-driven proliferation and migration in PDAC cells. (A) Representative colony formation images of PANC-1 cells transduced with control vector, E2F3 overexpression (OE-E2F3), or OE-E2F3 treated with the NF-κB inhibitor BAY 11-7082.
(B) Quantification of colony numbers. (C) Representative transwell migration images of PANC-1 cells under the same conditions. (D) Quantification of migrated cells. Data are presented as mean ± SD from three independent experiments. (E)Xenograft tumors from the experiment in Figure 7G ($n = 3$ per group) were collected and snap frozen for protein analysis. (F) Western blot validation of siTRIM26 knockdown efficiency in PANC-1 cells treated with two concentrations of siTRIM26 (20 nM and 50 nM) for 72 h.(G) Western blot analysis of TRIM26 expression in frozen tumor tissues from the therapeutic xenograft experiment shown in Figure 7J. Statistical analysis was performed using Student's *t*-test. **$p < 0.01$, ***$p < 0.001$; NS, not significant.

Table S1. Primary antibodies used in this study.

Target Protein	Vendor & Catalog #	Host	Dilution
NF-κB p65	CST #8242	Rabbit	1:1000
Phospho-NF-κB p65 (Ser536)	CST #3033	Rabbit	1:1000
IKKα	CST #2682	Rabbit	1:1000
IKKβ	CST #2684	Rabbit	1:1000
Phospho-IKKα/β (Ser176/180)	CST #2697	Rabbit	1:500
IκBα	CST #4812	Rabbit	1:1000
Phospho-IκBα (Ser32)	CST #2859	Rabbit	1:500
NF-κB2 p100	Abcam #ab175192	Rabbit	1:1000
Phospho-NF-κB2 p100 (Ser866/870)	CST #4810	Rabbit	1:500
E2F3	Abcam #ab320731	Rabbit	1:1000
TRIM26	Abcam #ab89290	Rabbit	1:1000
STYX	Abcam #ab205200	Rabbit	1:1000
MAP3K1	Proteintech #19970-1-AP	Rabbit	1:500
Phospho-MAP3K1	Proteintech #28844-1-AP	Rabbit	1:500
MEKK3	Abcam #ab40756	Rabbit	1:1000
Phospho-MEKK3 (Ser166)	Affinity #AF3545	Rabbit	1:500
TAK1	Abcam #ab109526	Rabbit	1:1000
Phospho-TAK1 (Ser412)	CST #9339	Rabbit	1:500
TAB1	CST #3226	Rabbit	1:1000
TRAF6	CST #8028	Rabbit	1:1000
Ubiquitin	Abcam #ab134953	Rabbit	1:1000
HA-Tag	Abcam #ab236632	Rabbit	1:5000
Flag-Tag	Abcam #ab205606	Mouse	1:5000
Myc-Tag	Abcam #ab32	Mouse	1:5000
Ki67	Abcam #ab16667	Rabbit	1:1000
GAPDH	CST #2118	Mouse	1:5000
Secondary antibodies	Boster#BM2006	Goat	1:5000

Table S2. Primer sequences used for RT-qPCR analysis.

NAME	Forward Primer (5'→3')	Reverse Primer (5'→3')
E2F3(NM_001949/E2F3A)	ATGAGAAAGGGAATCCAGCCCG	GGTGGTGGAAGTGTTCGTGGT
E2F3(NM_001243076/E2F3B)	ATGCCCTTACAGCAGCAGGC	TGGTGAGCAGACCAAGAGACG
TRIM26	ATGGCCACGTCAGCCCCACT	TCAGGGTCTTAGCAGGAGGC
MYC		
CCND1		
IL6	ATGAACTCCTTCTCCACAAGC	CTACATTTGCCGAAGAGCC
CXCL8/IL8	ATGAACGGCAAACTTGGGGTTGTC	TTACAGCGGTGCATCAGAATTGAGC
TNF		
IL1B	ATGGCAGAAGTACCTGAGCT	TTAGGAAGACACAAATTGCATGGTG
GAPDH		

Table S3. shRNA and siRNA sequences used for gene silencing.

NAME	F	R
shTRIM26_1	CCGGGCAGTACATTGTGGCGGAATTCTCGAGAATTCCGCCACAATGTACTGCTTTTTG	AATTCAAAAAGCAGTACATTGTGGCGGAATTCTCGAGAATTCCGCCACAATGTACTGC
shTRIM26_2	CCGGGCCATCCCTCACATGGTTAAACTCGAGTTTAACCATGTGAGGGATGGCTTTTTG	AATTCAAAAAGCCATCCCTCACATGGTTAAACTCGAGTTTAACCATGTGAGGGATGGC
siSTYX	GAGGCCCAGAGTGTGTATACC	
shE2F3_1	CCGGCCCGCTTTACTCTTCAGGAATCTCGAGATTCCTGAAGAGTAAAGCGGGTTTTTG	AATTCAAAACCCGCTTTACTCTTCAGGAATCTCGAGATTCCTGAAGAGTAAAGCGGG
shE2F3_2	CCGGCCCGCTTTACTCTTCAGGAATCTCGAGATTCCTGAAGAGTAAAGCGGGTTTTTG	AATTCAAAACCCGCTTTACTCTTCAGGAATCTCGAGATTCCTGAAGAGTAAAGCGGG
SiE2F3	CCCGCTTTACTCTTCAGGAAT	

Table S4. ChIP-qPCR primer sequences used for TRIM26 promoter occupancy assays.

name	F Sequence (5'→3')	R Sequence (5'→3')
Primer1	AGAGTTGGGCAGTGAGGGGT	GAAATCAGGCGGGACCGAGG
Primer2	CAAGTCTCGGCCCGCTTTGT	AATACGAAGTCCCCGCCCCT
Primer3	CTCGGCTCACTGCAACCTCC	GGGCGGATCACGAGGTCAAG
Primer4	ATTTCCCTACCCCCAGCGGA	GCCAGTGCCCTGCTCCTAAG

Table S5. Primers for TRIM26 promoter cloning and site-directed mutagenesis.

Use	Primer ID	Sequence (5'→3')
Promoter cloning (−400 to +100)	TRIM26-promoter-KpnI-F	TAAGCAGGTACCGCGCCCTCGGACCCTGA
Promoter cloning (−400 to +100)	TRIM26-promoter-HindIII-R	TGCTTAAAGCTTGGTCCCGAGCTCCGGG
Site-directed mutagenesis (E2F core)	TRIM26-E2F-mut-F	ACtattaAAACTCAGGCTGCGGCTCTTGGCGC
Site-directed mutagenesis (E2F core)	TRIM26-E2F-mut-R	GCCTGAGTTTtaataGTAATTGGGGTGGCCTGTTACA
